# Supplementary material for: Fully automated fast-flow synthesis of antisense phosphorodiamidate morpholino oligomers
Source: Nat Commun. 2021 Jul 20;12:4396. doi: 10.1038/s41467-021-24598-4 (PMC8292409; doi:10.1038/s41467-021-24598-4)
Supplement: Supplementary file 1 — Supplementary Information [file 41467_2021_24598_MOESM1_ESM.pdf]

Supplementary Information for

Fully Automated Fast-Flow Synthesis of Antisense Phosphorodiamidate Morpholino Oligomers

Authors:

Chengxi Li[1†], Alex J. Callahan[1†], Mark D. Simon[1], Kyle A. Totaro[1], Alexander J. Mijalis[1], Kruttika-Suhas Phadke[2], Genwei Zhang[1], Nina Hartrampf[1,3], Carly K. Schissel[1], Ming Zhou[4], Hong Zong[4], Gunnar J. Hanson[4], Andrei Loas[1], Nicola L. B. Pohl[5], David E. Verhoeven[2], Bradley L. Pentelute[1,6,7,8*]

Affiliations:

[1]Department of Chemistry, Massachusetts Institute of Technology, 77 Massachusetts Avenue, Cambridge, MA 02139, USA.

[2] Department of Veterinary Microbiology and Preventive Medicine, College of Veterinary Medicine, Iowa State University, Ames, IA 50011 USA.

[3]Current address: University of Zurich, Department of Chemistry, Winthertkurerstrasse 190, 8057 Zurich, Switzerland.

[4]Sarepta Therapeutics, 215 First Street, Cambridge, MA 02142, USA.

[5]Department of Chemistry, Indiana University, 120A Simon Hall, 212 S. Hawthorne Drive, Bloomington, IN 47405, USA.

[6]The Koch Institute for Integrative Cancer Research, Massachusetts Institute of Technology, 500 Main Street, Cambridge, MA 02142, USA.

[7]Center for Environmental Health Sciences, Massachusetts Institute of Technology, 77 Massachusetts Avenue, Cambridge, MA 02139, USA.

[8]Broad Institute of MIT and Harvard, 415 Main Street, Cambridge, MA 02142, USA.

*E-mail: blp@mit.edu

[†]These authors contributed equally to this work.

This PDF file includes:

Materials and Methods
Supplementary Text
Figs. S1 to S21

CONTENTS

1. GENERAL INFORMATION 4
1.1 GENERAL REAGENT INFORMATION 4
1.2 LC-MS ANALYSIS 5
1.3 MALDI ANALYSIS 6
1.4 HPLC ANALYSIS 6
2. PMO SYNTHESIS REAGENTS 7
2.1 BATCH REAGENTS 7
2.1.1 Detritylation solution 7
2.1.2 Neutralization solution 7
2.1.3 Coupling solution 7
2.2 FLOW REAGENTS 7
2.2.1 Detritylation solution stock 7
2.2.2 Neutralization solution stock 8
2.2.3 Monomer solution stock 8
2.2.4 Coupling base stock 8
3. PROCEDURE FOR BATCH REACTIONS OF PMO 8
3.1 RESIN LOADING WITH EG3 TAIL 8
3.2 BATCH SYNTHESIS OF PMOS 8
3.2.1 Resin Preparation 8
3.2.2 Deprotection 8
3.2.3 Neutralization 9
3.2.4 Coupling 9
4. CLEAVAGE METHODS 11
5. PURIFICATION METHODS 11
5.1 CATION EXCHANGE PURIFICATION 11
5.2 REVERSE PHASE PURIFICATION 12
5.3 ANION EXCHANGE PURIFICATION 12
6. QUANTIFICATION OF 4-MER SYNTHESIS FROM LC-MS DATA 12
7. HIGH BOILING POINT SOLVENT SCREEN FOR BATCH SYNTHESIS 13
8. MONOMER THERMAL STABILITY STUDIES 15
9. RESIN BOUND PMO THERMAL STABILITY STUDIES 17
9.1 STABILITY IN THE SYNTHETIC SOLVENT AT 90°C 17
9.2 STABILITY WITH AND WITHOUT NEUTRALIZATION AT 90°C 18

9.3 STABILITY TO INCUBATION IN DCE AT 70°C AND 90°C 19
9.4 STABILITY TO BATCH DETRITYLATION CONDITIONS AT 70 °C AND 90 °C 20
9.5 DETRITYLATION OPTIMIZATION AT 90 °C 20

10. DESIGN OF A FULLY AUTOMATED FLOW SYNTHESIZER 23

10.1 GENERAL INTRODUCTION OF TINY TIDES 23
10.2 REACTOR DESIGN 25
10.3 POSITIVE NITROGEN PRESSURE 26
10.4 MIXER DESIGN 28
10.5 UV-VIS MONITORING 29
10.6 PUMP HEAD CAVITATION 30

11. PMO CHAIN STABILITY UNDER OPTIMIZED CONDITIONS 32

12. OPTIMIZED AUTOMATED SYNTHESIS SEQUENCE 33

13. RAW LCMS DATA 34

13.1 LCMS TRACES OF 4-MER PMOS FOR CONDITION OPTIMIZATION 34
13.2 LCMS DATA OF 12-MER WITH TINY TIDES 70˚C SYNTHESIS 47
13.2.1 Batch synthesis of 12-mer PMO 47
13.2.2 Tiny Tides synthesis of 12-mer PMO at 70˚C 48
13.3 LCMS DATA OF 18-MER (IVS2-654) 90˚C SYNTHESIS 49
13.3.1 Batch synthesis of 18-mer PMO (IVS2-654) (crude) 49
13.3.2 Batch synthesis of 18mer PMO (IVS2-654) (post purification) 50
13.3.3 Tiny Tides synthesis of 18-mer PMO (IVS2-654) at 90˚C 51
13.3.4 Tiny Tides synthesis of 18-mer PMO (IVS2-654) at 90˚C (post purification) 52
13.4 LCMS DATA OF EXON 46 TARGETED 20-MER SEQUENCES 53
13.4.1 Tiny Tides synthesis of 20-mer PMO (seq. 1) at 90˚C 54
13.4.2 Tiny Tides synthesis of 20-mer PMO (seq. 2) at 90˚C 54
13.4.3 Tiny Tides synthesis of 20-mer PMO (seq. 3) at 90˚C 56
13.5 LCMS DATA OF SARS-COV-2 5’UTR TRS TARGETED SEQUENCE 57
13.6 LCMS DATA OF PURIFIED FUSION INHIBITOR EK1 58

14. REFERENCES 59

1. General Information

1.1 General reagent information

Activated morpholino subunits, moA, moC, moG, and moT (Supplementary Figure 1), functionalized aminomethyl polystyrene resin (Supplementary Figure 2), and functionalized polyethylene glycol linker ("Tail", Supplementary Figure 3) were all provided by Sarepta Therapeutics. 1,3-dimethyl-2-imidazolidinone (DMI), N-methyl-2-pyrrolidone (NMP), dichloromethane (DCM), and N,N-diisopropylethylamine (DIEA) were obtained anhydrous from Sigma-Aldrich (St. Louis, MO). HPLC-grade Acetonitrile was purchased from VWR International (Philadelphia, PA) and LC-MS grade acetonitrile was purchased from Sigma-Aldrich (St. Louis, MO). Econo-Pac chromatography columns and accessories for solid-phase extraction were purchased from Bio-Rad (Hercules, CA). Water for HPLC was purified to 18.2MΩ·cm resistivity on a Millipore Milli-Q system. All other reagents and solvents were purchased from Sigma-Aldrich (St. Louis, MO) as the purest anhydrous grades available, and used without further purification. Unless specified otherwise, all solvents used were kept over activated 3 Å molecular sieves.

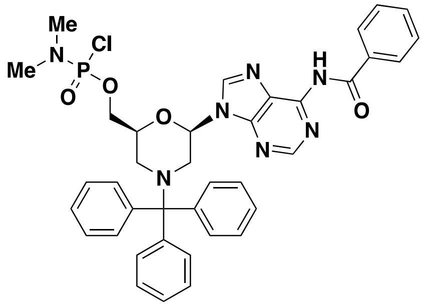

Activated, protected adenine monomer "moA"

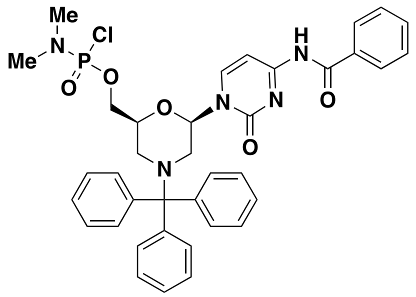

Activated, protected cytosine monomer "moC"

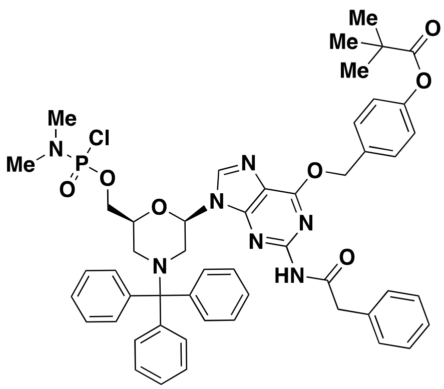

Activated, protected guanine monomer "moG"

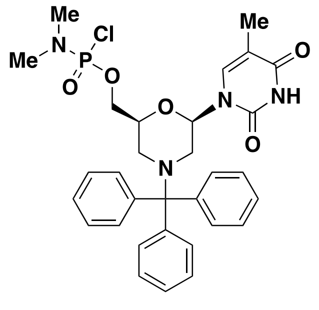

Activated, protected thymine monomer "moT"

Supplementary Figure 1: Structures of morpholino subunits

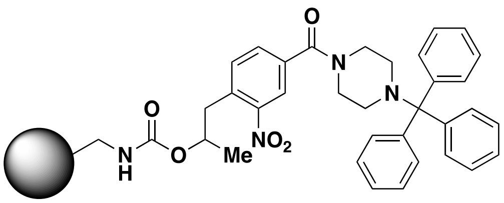

Functionalized, protected polystyrene resin
0.39-0.43 mmol/g

Supplementary Figure 2: Structure of functionalized polystyrene resin

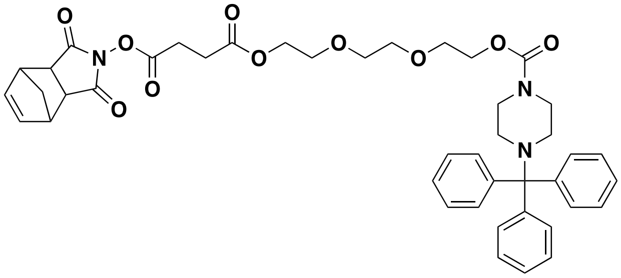

Activated, protected polyethylene glycol linker
"Tail"

Supplementary Figure 3: Structure of the activated polyethylene glycol "Tail"

1.2 LC-MS analysis

Condition 1: Analysis was performed on an Agilent 1290 Infinity HPLC coupled to an Agilent 6520 ESI-Q-TOF mass spectrometer. MS was run in positive ionization mode, extended dynamic range (2GHz), and standard mass range (m/z in range 300 to 3000). The solvent mixtures used for LC-MS chromatography were: A = water + 0.1% formic acid (LC-MS-grade), B = acetonitrile + 0.1% formic acid (LC-MS-grade). The following condition was used for PMO analysis. Column: Zorbax 300-SB C3 (5 μm, 150 x 2.1 mm, 300Å silica); Flow Rate: 0.8 mL/min; Gradient: 1% B 0-2 min, linearly ramp from 1% B to 61% B 2 to 11 min, 61% B to 95% B 11 to 12 min. Post time is 1% B for 3 min. Flow rate is 0.8 mL/min. MS data was acquired from 4 to 11 minutes.

Condition 2: Analysis was performed on an Agilent 1290 Infinity HPLC coupled to an Agilent 6550 Q-TOF with Dual Jet Stream ESI ionization and iFunnel. MS was run in positive ionization mode, extended dynamic range (2GHz), and low mass range (m/z in range 100 to 1700). The solvent mixtures used were as above. Column: Phenomenex Luna C18 (2) (3 μm, 150 x 1 mm, 100 Å silica); Flow Rate: 0.05 mL/min; Gradient: 1% B 0-2 min, linearly ramp from 1% B to 61% B 2 to 14 min, 61% B. Post time is 1% B for 3 min. MS data was acquired from 4 to 14 minutes.

Condition 3: Agilent 6550 (1290 Infinity HPLC system with Dual Jet Stream ESI ionization followed by iFunnel Q-TOF MS. MS is run in positive ionization mode, extended dynamic range (2GHz), and standard mass range (m/z in range 300 to 3000). The solvent mixtures used were as above Column: Phenomenex Kinetex PS C18 (100 Å, 2.6 μm, 2.1 x 100 mm); Flow Rate: 0.4 mL/min; Gradient: 0% hold for 3 minutes, 0-60% B over 67 minutes, MS on from 2-62 minutes.

Condition 4: Agilent 6550 (1290 Infinity HPLC system with Dual Jet Stream ESI ionization followed by iFunnel Q-TOF MS. MS is run in positive ionization mode, extended dynamic range (2 GHz), and standard mass range, m/z in range 300 to 3000). The solvent mixtures used were as above Column: Phenomenex Kinetex PS C18 (100 Å, 2.6 μm, 2.1 x 100 mm); Flow Rate: 0.4 mL/min; Gradient: 0% hold for 3 minutes, 0-60% B over 17 minutes, MS on from 2-15 minutes.

1.3 MALDI analysis

All MALDI spectra were collected on a Bruker Microflex II in linear mode, with positive ionization in low mass range (3000-8000 m/z range) and laser power under 50%. 1 μL of each sample was deposited onto a ground-steel MALDI target plate, mixed with 1 μL saturated sinapinic acid in 50% aqueous acetonitrile, and allowed to air dry.

1.4 HPLC analysis

Analytical HPLC was carried out on an Agilent 1200 series system with UV detection at 260 nm. Column: Zorbax CN SB, (150 x 4.6 mm, 3.6 μm, 100 Å silica); flow rate 0.8 mL/minute; Solvent System: A = water with 10 mM triethylamine acetate buffer pH 5.65, B = acetonitrile; Gradient: 3 minute hold 5% B, 5-65% B gradient over 60 minutes, 3 minute hold 65% B, 10 minute post run 5% B.

2. PMO synthesis reagents

2.1 Batch Reagents

The following is a list of the reagents used for batch PMO synthesis.

2.1.1 Detritylation solution

100 mM 4-cyanopyridine trifluoroacetate in 4:1 (v/v) DCM to trifluoroethanol (TFE) + 1% (v/v) ethanol. The solution was made by first mixing the DCM and TFE, followed by sequential addition of 4-cyanopyridine, trifluoroacetic acid (TFA), and ethanol. Solution was prepared fresh and not stored for more than 2 days.

2.1.2 Neutralization solution

5% DIEA in 3:1 DCM/isopropanol (*i*PrOH) (v/v).

2.1.3 Coupling solution

0.2 M morpholino subunit, 0.4 M DIEA, and 0.2 M LiBr in DMI. The solution was made by first dissolving the subunit and LiBr in DMI. Base was added immediately prior to coupling.

2.2 Flow Reagents

The following is a list of the stock solutions that were used for optimized flow PMO synthesis. During automated flow synthesis, reaction mixtures were diluted two fold prior to reaching the resin bed so the stocks were more concentrated than the corresponding batch synthesis solutions.

2.2.1 Detritylation solution stock

For 70˚C synthesis: 400 mM 4-cyanopyridine trifluoroacetate in 3:2 (v/v) DCM/TFE + 2% (v/v) ethanol. The solution was made by first mixing the DCM and TFE, followed by sequential addition of 4-cyanopyridine, TFA, and ethanol. Solution was prepared fresh and stored under dry nitrogen pressure and used the same day.

For 90˚C synthesis: 800 mM 3,5-lutidine trifluoroacetate in 3:2 (v/v) DCM/TFE + 2% (v/v) ethanol. The solution was made by first mixing the DCM and

TFE, followed by sequential addition of 3,5-lutidine, TFA, and ethanol. Solution was prepared fresh and stored under dry nitrogen pressure and used the same day.

2.2.2 Neutralization solution stock

10% NEM in 1:1 DCM/*i*PrOH (v/v).

2.2.3 Monomer solution stock

0.4 M morpholino subunit in DMI.

2.2.4 Coupling base stock

0.8 M DIEA, and 0.4 M LiBr in DMI.

3. Procedure for batch reactions of PMO

3.1 Resin loading with EG3 Tail

Aminomethyl polystyrene resin used in all studies had a loading of 0.39-0.43 mmol/g and was functionalized with the "Tail" PEG linker bearing a piperazine terminus, according to literature procedres[1].

3.2 Batch synthesis of PMOs

A procedure for the batch synthesis of PMOs was adapted from patent and academic reports[1-5]. Each step is described below (Supplementary Figure 4).

3.2.1 Resin Preparation

100 mg of tail-loaded aminomethyl polystyrene resin (0.39-0.43 mmol/g loading) (39-43μmol) was placed in a 20-mL disposable reaction vessel (Torviq) and swollen in NMP for at least 1 hour.

3.2.2 Deprotection

Resin was washed with 30% TFE in DCM (2 x 7 mL) and placed in 7 mL of **Detritylating solution** (**Supplementary Section 2.1.1**) for 15 minutes. This process was repeated 2 times.

3.2.3 Neutralization

Deprotected resin was washed with DCM (2 x 7 mL) and 30% TFE in DCM (2 x 7 mL), and then placed in 7 mL of **Neutralization solution** (**Supplementary Section 2.1.2**) for 5 minutes. This process was repeated 2 times.

3.2.4 Coupling

Neutralized resin was washed with DCM (2 x 7 mL), 30% TFE in DCM (2 x 7 mL), and DMI (2 x 7 mL). 10 eq. of monomer relative to the highest resin loading was added as **Coupling solution** (**Supplementary Section 2.1.3**) and reacted for 90 minutes. Resin was then washed with DCM (3 x 7 mL) and then placed in 7 mL of **Neutralization solution** (**Supplementary Section 2.1.2**) for 5 minutes. This process was repeated 2 times. Finally, resin was washed with DCM (3 x 7 mL) and 30% TFE in DCM (3 x 7 mL).

(a)

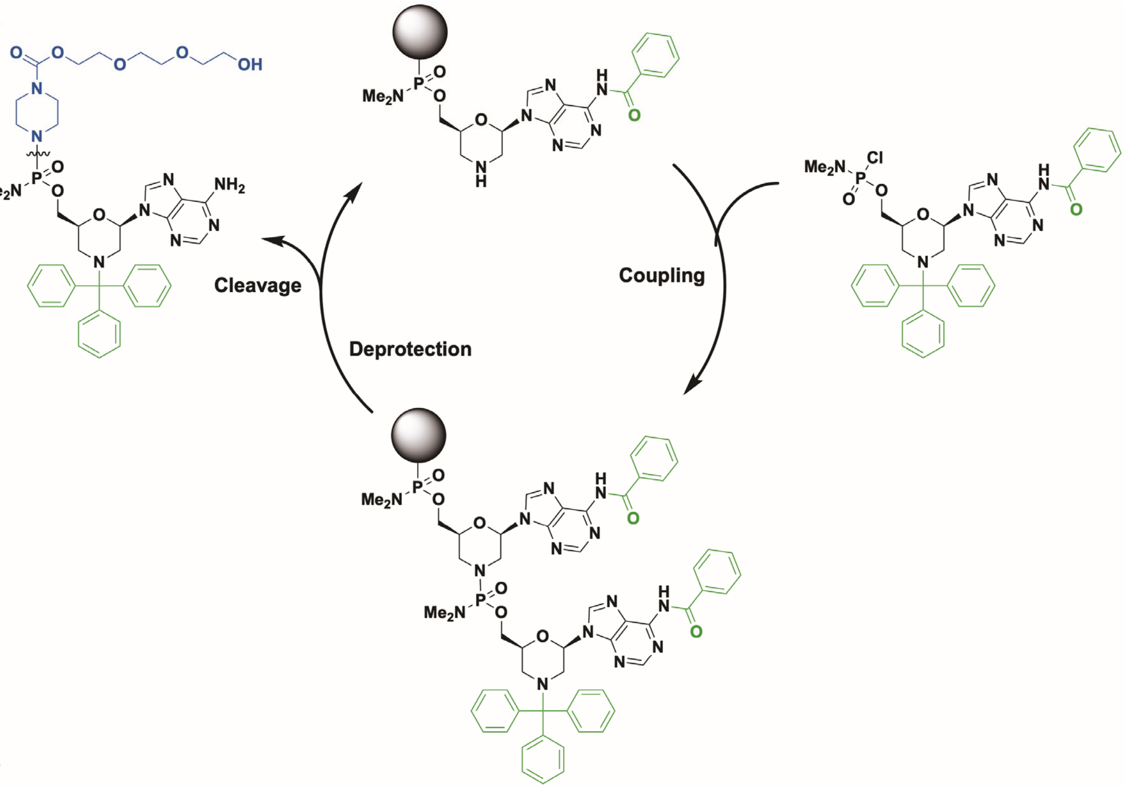

(b)

Entry	Step	Volume	Time	Mixture
**1**	Wash	14 mL	15 sec	30% TFE:DCM

**2**	Detritylation	7 mL	15 min	100 mM 4-cyanopyridine 100 mM TFA in 4:1 DCM:TFE, 1% EtOH
**3**	Detritylation	7 mL	15 min	100 mM 4-cyanopyridine 100 mM TFA in 4:1 DCM:TFE, 1% EtOH
**4**	Wash	14 mL	15 sec	DCM
**5**	Wash	14 mL	15 sec	30% TFE:DCM
**6**	Neutralization	7 mL	5 min	5% DIEA in 3:1 DCM:*i*PrOH
**7**	Neutralization	7 mL	5 min	5% DIEA in 3:1 DCM:*i*PrOH
**8**	Wash	14 mL	15 sec	DCM
**9**	Wash	14 mL	15 sec	30% TFE:DCM
**10**	Wash	14 mL	15 sec	DMI
**11**	Coupling	2.6 mL	90 min	0.18 M monomer, 0.4 M DIEA, 0.2 M LiBr in DMI
**12**	Wash	21 mL	30 sec	DCM
**13**	Neutralization	7 mL	5 min	5% DIEA in 3:1 DCM:*i*PrOH
**14**	Neutralization	7 mL	5 min	5% DIEA in 3:1 DCM:*i*PrOH
**15**	Wash	21 mL	30 sec	DCM
**16**	Wash	21 mL	30 sec	30% TFE:DCM
**Total**	--	192 mL	143 min	--

Supplementary Figure 4: The procedure for manual PMO synthesis is shown*. a) The chemical transformations that take place during PMO synthesis are shown. Green structures represent protecting groups that are lost upon cleavage. Blue structures represent the PEG$_3$ tail appended at the 5' end of each PMO sequence. Each nucleotide is incorporated as a trityl protected phosphoramido chloridate. Detritylation and neutralization regenerate a reactive 3' amine for the next coupling reaction. b) The manual procedure used to produce PMOs in batch is shown.*

4. Cleavage methods

Completed PMO resins were washed with DCM and dried under vacuum prior to cleavage. Crude resin was placed in a 7 mL glass vial with a tight-fitting Teflon-lined screw cap and cleaved using one of the following methods:

Method 1: A 1:1 mixture of ammonia (sat. aq.) and methylamine (sat. aq.) was freshly prepared and 1 mL per 10 mg of crude resin was added. The vial was capped then kept at 65 °C for 15 minutes.

Method 2: A 1:1 mixture of ammonia (sat. aq.) and ethanol was freshly prepared and 1 mL per 10 mg of crude resin was added. The mixture vial was capped then kept at 65 °C for 36 hours.

The entire crude cleavage mixture was filtered through a 0.22 μm PTFE syringe filter and washed with 3 x 1 mL methanol. The filtrate was evaporated to dryness using nitrogen flow.

The residue was dissolved in 10 mL of Milli-Q water prior to SPE. Separately, SPE columns were prepared and conditioned following a previously reported procedure[1]. 20 mL Econo-Pac columns from Bio-Rad were charged with 3-4 mL of Amberchrome CG-300M resin and sealed with a frit. Then 8 mL of the following solutions were added to the column, in order, and drained before adding the next solution: 80% ACN in 1% NH_4OH (sat. aq.), 0.5 M NaOH in 20% EtOH, water, 50 mM H_3PO_4 in 80% ACN, water, 0.5 M NaOH in 20% EtOH, water, 1% NH_4OH (sat. aq.). When conditioning was complete, the column was stored in 8 mL of 1% NH_4OH (sat. aq.) at room temperature until used. The column was rinsed two times with 12 mL of water before loading the crude mixture onto the column. Then, the column was rinsed once with 3 mL of 1 M NaCl, followed by three rinses with 12 mL of water, and once with 3 mL of 10% acetonitrile in water. The PMO was then eluted with two 3 mL rinses of 50% acetonitrile in water. The eluent from the 50% acetonitrile wash was collected into a pre-weighed 15 mL conical centrifuge tube and lyophilized to afford the crude PMO as a white powder suitable for LC-MS analysis and purification.

5. Purification methods

5.1 Cation Exchange Purification

Method 1: Analytical cation exchange HPLC purification was carried out on an Agilent 1200 series system with UV detection at 260 nm. Column: ProPac SCX-20

(20 μM, 4 x 250 mm); Flow rate 1.0 mL/minute; Solvent system: C = 20 mM KH_2PO_4, pH 2.0 in water with 20% acetonitrile, D = solvent C with 1 M KCl; Gradient: 5 minute hold 0% D, 0-50% D linear gradient over 100 minutes, 50-70% D over 3 minutes, 3 minute hold 70% D, 10 minute post run 0% D; Flow rate: 1.0 mL/minute. Fractions collected from 5 to 105 minutes with 1 minute time slices.

Method 2: Purification was carried out on an ÄKTA Pure using a 5 mL HiTrap SP HP column with UV detection at 260 nm; Flow rate: 5.0 mL/minute; Solvent system: E = 20% acetonitrile in water with 10 mM phosphoric acid, F = solvent E with 1 M potassium chloride; Gradient: 5 minute hold at 0% F, 0-10% F over 10 minutes, 10-40% F linear gradient over 120 minutes, 0% F for 10 minutes. Fractions collected from 10 to 120 minutes with 1 minute time slices.

5.2 Reverse Phase Purification

Analytical HPLC purification was carried out on an Agilent 1200 series system with UV detection at 260 nm. Column: Zorbax CN SB (150 x 4.6 mm, 3.6 μm, 100 Å silica); Flow rate: 0.8 mL/minute; Solvent system: G = water with 10 mM triethylamine acetate buffer, pH 5.65, H = acetonitrile; Gradient: 3 minute hold 5% H, 5-15% H linear gradient over 10 minutes, 15-35% H linear gradient over 100 minutes, 35-65% H linear gradient over 3 minutes, 3 minute hold 65% H, 10 minute post run 5% H; Flow rate: 0.8 mL/minute. Fractions collected from 15 to 115 minutes with 1 minute time slices.

5.3 Anion Exchange Purification

Purification was carried out on an ÄKTA pure using a 5 mL HiTrap Q HP column with UV detection at 260 nm; Flow rate 5.0 mL/minute; Solvent system: I = water with 10 mM sodium hydroxide, J = water with 10 mM sodium hydroxide and 1 M sodium chloride; Gradient: 5 minute hold at 0% J, 0-10% J linear gradient over 10 minutes, 10-40% J linear gradient over 120 minutes, 0% J for 10 minutes. Fractions collected from 10 to 120 minutes with 1 minute time slices.

6. Quantification of 4-mer synthesis from LC-MS data

Side products from PMO synthesis often co-elute on RP-HPLC, so quantification of sample purity was measured using total extracted ion counts. Extracted ion counts were tabulated using Agilent's Molecular Feature Extraction utility. For each sample, all tabulated ions that arose from the intended PMO sequence were summed. Small amounts of side products that didn't arise from the

synthesis process, but instead arose from the cleavage process, or the purification method itself were included in the summations as pure product. For each species quantified, the abundances of the following identified compounds were summed: [M+14], [M+Bz protecting group], [M+Bz protecting group+14], and [M+G base elimination]. [M+14] is caused by methylation of cytosine residues from cleavage in methylamine. [M+Bz protecting group] is the result of incomplete removal of benzoyl protecting groups on A and C residues. [M+G base elimination] is a result of loss of the G nucleobase that occurs under the acidic conditions in the LC-MS separation. Not included in the analyses are small molecules that are arise from the cleavage process. To ensure that these molecules were excluded from the calculation, the extraction utility ignored compounds with molecular weight less than 500 Da, and avoided peaks of the LC-MS separation that resulted from known cleavage molecules.

The following parameters were used in the Molecular Feature Extraction utility: Find by Molecular Feature; Restrict m/z to 500-2000; Restrict retention time to 6.5-8.0 minutes (this time range covers desired product and structure related side-products); No mass filter; No mass defect; Use peaks with height >= 100; Standard ion species; Limit max number of peaks to 100; Isotope spacing tolerance 0.0025 m/z plus 7.0 ppm; Isotope model: Common organic molecules; Limit assigned charge stated to a maximum of 3.

7. High boiling point solvent screen for batch synthesis

We investigated solvents to replace DCM so as to avoid the difficulties of working with solvents over their boiling point. DCM is the preferred solvent for washes and detritylation, while either DMI or NMP are preferred coupling solvents. The latter solvents were appropriate for flow synthesis at high temperatures, but the atmospheric pressure boiling point of DCM is only 42 °C. We sought to improve process safety and reliability by substituting a higher boiling solvent for DCM during detritylation.

To screen high boiling solvents for PMO synthesis, a modified, small-scale (4 μmol) room temperature batch synthesis protocol was used with the same basic outline as the procedure outlined in **Supplementary Section 3**. Couplings were only 15 minutes using 25 eq. (0.1 mmol) of monomer (Supplementary Figure 5). The product was left Trityl protected at the end of synthesis. This abbreviated batch protocol afforded the model 4-mer PMO 5'-ACGT-Trityl-3' with minor deletion products. Tracking relative amounts of these by-products enabled comparison between reaction solvents.

Alternative solvents were investigated by repeated synthesis using the modified procedure. To examine the effect of detritylation solvents, each

detritylation was performed with 100 mM 4-cyanopyridine trifluoroacetate, 1% (v/v) ethanol, and 20% (v/v) trifluoroethanol dissolved in DCM (Control), dichloroethane (DCE), toluene, dioxane, acetonitrile, diglyme, NMP or dimethylformamide (DMF) (Supplementary Figure 5). All couplings in these experiments were performed in DMI. The products from each of these syntheses were examined by LC-MS and DCM, DCE, and toluene were found to be suitable detritylation solvents, with DCE slightly outperforming the DCM control.

(a)

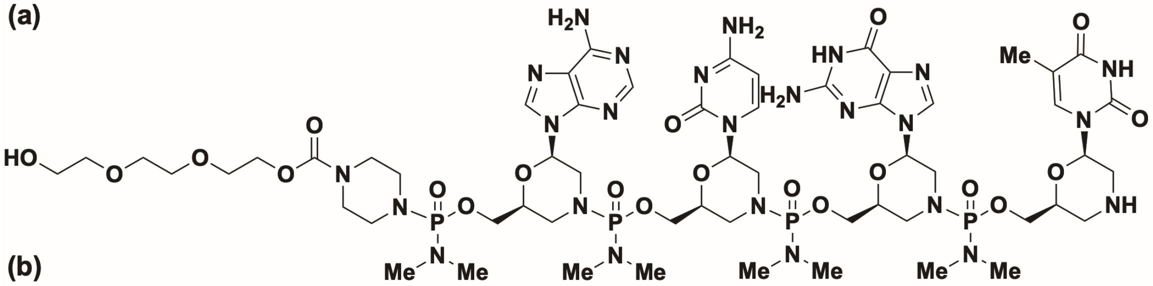

(b)

Entry	Coupling solvent	Detritylation solvent	Temperature (˚C)	Purity (%)	C-Deletion	G-Deletion
**1**	DMI	DCM	r.t.	88	0.5	2
**2**	DMI	DCE	r.t.	91	<0.5	<0.5
**3**	DMI	Toluene	r.t.	84	<0.5	<0.5
**4**	DMI	Dioxane	r.t.	Failed	**	**
**5**	DMI	Acetonitrile	r.t.	Failed	**	**
**6**	DMI	Diglyme	r.t.	Failed	**	**
**7**	DMI	NMP	r.t.	Failed	**	**
**8**	DMI	DMF	r.t.	Failed	**	**
**9**	DCE	DCE	r.t.	86	<0.5	3
**10**	Toluene	DCE	r.t.	47	1.5	34
**11**	NMP	DCE	r.t.	87	<0.5	<0.5
**12**	DMF	DCE	r.t.	87	0.5	1

***Supplementary Figure 5: Solvent screening at room temperature identifies high-boiling solvents for PMO synthesis**. a) The structure for the synthesized 4-mer PMO is shown. b) Purity values were extracted from LC-MS data as described in **Supplementary Section 6**. Samples were run with standard LCMS method **Supplementary Section 1.2 Condition 1**. A and T deletions were very low intensity or unidentified; the last 10-20% of assigned ions were unknown species.*

To select between DCE and toluene as the detritylation solvent, these were evaluated as coupling solvents along with DMI (control), NMP, and DMF. In each of 5 experiments, every coupling in the model tetramer was carried out with 0.2 M monomer and 0.4 M NEM in one of these solvents (Supplementary Figure 5). DCE performed the best during the coupling trials, indicating residual DCE would have minimal effect on coupling, so it was selected as the detritylation solvent for the current stage.

Ultimately, however, the resin bound PMO was found to be unstable in DCE at elevated temperature, possibly due to elimination of hydrogen chloride and subsequent acid catalyzed chain decomposition (**Supplementary Section 9.2**). Although additional high boiling solvents could be screened, we ultimately decided to use DCM above its normal boiling point. The flow instrument reported here has little trouble handling DCM at 90 ˚C where it's vapor pressure is only about 5 bar.

8. Monomer thermal stability studies

The thermal stability of the activated phosphoramido chloridate monomers used for coupling, determined 90 °C as the maximum temperature for flow synthesis. While not necessary for room temperature procedures, anhydrous conditions are required at 90 °C. Of particular interest was the propensity for the monomers to decompose via polymerization.

To determine the maximum allowed temperature for a flow synthesis, we assayed the thermally induced degradation of each monomer (moA, moT, moC, moG) at gradually increasing temperature. In sealed vials, 0.2 M coupling solutions of activated, protected morpholino monomer and 0.4 M *N,N*-diisopropylethylamine (DIEA) in 1,3-dimethyl-2-imidazolidinone (DMI) were heated for 5 minutes at temperatures from 90 °C to 150 °C (Supplementary Figure 6). After 5 minutes, coupling solutions were quenched with a 10% (v/v) solution of piperidine in *N*-methyl-2-pyrrolidone (NMP) and analyzed by liquid chromatography-mass spectrometry (LC-MS). Degradation was characterized by the disappearance of the major peak of the piperidine-quenched monomers and the increase in uncharacterized side-product peaks. Stability was evaluated over 5 minutes because this was the predicted residence time following rapid preheating in a flow system. The moA monomer degraded to unknown products at higher temperatures (Supplementary Figure 6).

The thermal stability of the monomers moT, moC, and moG was not investigated at temperatures over 90°C. The monomer moA degraded at temperatures over 90°C, invalidating any conditions at temperatures over this limit.

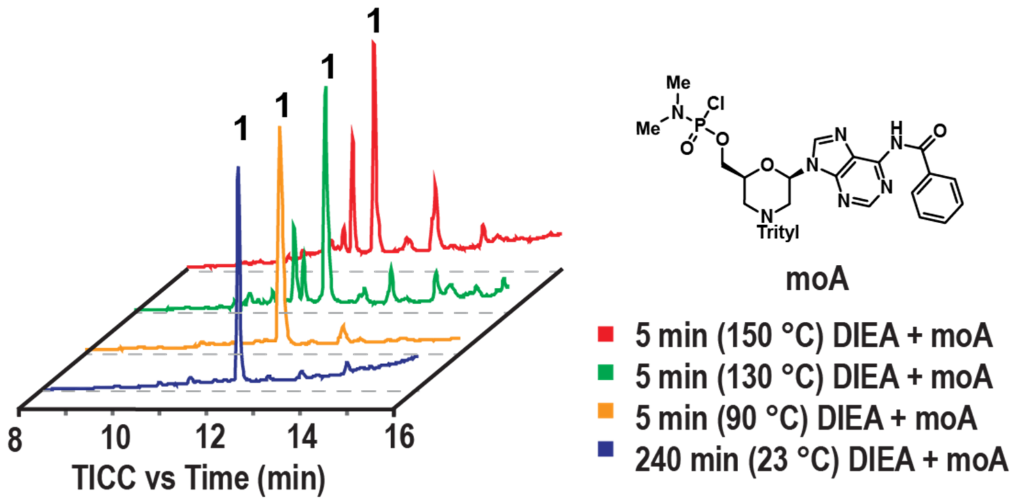

Supplementary Figure 6: *The activated PMO monomer for the nucleobase adenine is stable for 5 minutes at 90 °C. Higher temperatures lead to degradation of the activated monomer species. Peak 1 in the LCMS trace represents the remaining active monomer, and as temperature is increased, peak 1 diminishes compared to degradation products.*

Monomer stability was examined at 90°C by heating coupling solutions of 0.2 M moA and 0.4 M DIEA in DMI in sealed vials for 5 minutes with or without LiBr. After 5 minutes, coupling solutions were quenched with a 10% (v/v) solution of piperidine in NMP and analyzed by LC-MS (Supplementary Figure 7). All monomers did not display any degradation products.

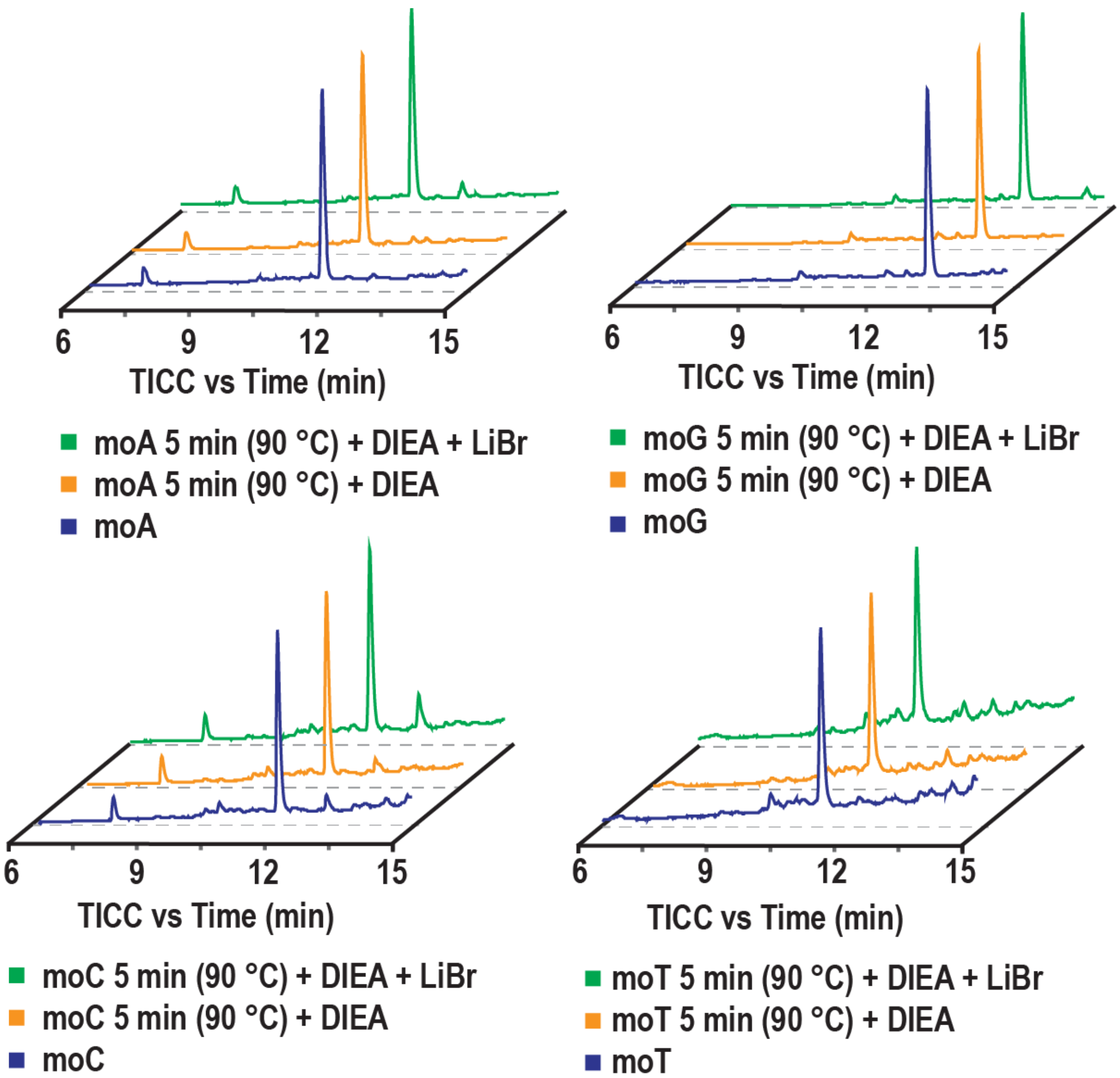

Supplementary Figure 7: All four monomers tested were stable for 5 minutes at 90°C*. Monomer solutions were quenched with piperidine and analyzed by LC-MS as in* ***Supplementary Section 1.2 Condition 1****. The major peaks in all conditions correspond to the piperidine quenched monomer.*

9. Resin bound PMO thermal stability studies

9.1 Stability in the synthetic solvent at 90°C

Although we were unable to find any reports of instability of the resin-bound protected PMO chain in the standard wash solvents, dichloromethane (DCM), DMI, and neutralization solution made from 5% 4-ethylmorpholine (NEM) in 3:1 DCM to isopropanol (v/v), we investigated this behavior at 90 °C. A protected resin-bound PMO with the sequence 5’-CTCCAACATC-3’ was treated with these solvents at elevated temperatures for 4 hours. For most conditions tested, 90 °C was chosen

to emulate the maximum possible temperature from the monomer studies. The only exception to this was DCM which was performed at 70 °C. The lower temperature was chosen to reduce pressure in the glass vials used. The 4-hour treatment time was selected since it is an excess of the time required for a typical flow synthesis. The resin was isolated, washed, cleaved, and degradation was measured using LC-MS. Protected PMO attached to a solid support does not degrade at 90 °C in the solvents used for room temperature syntheses (Supplementary Figure 8).

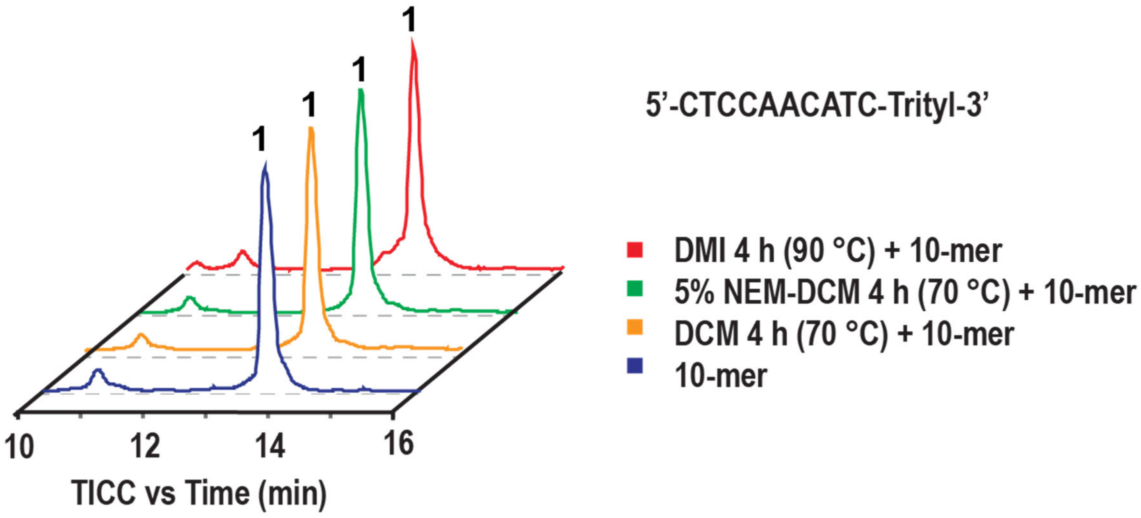

Supplementary Figure 8: *Resin-bound 10-mer PMO is stable to 90 °C incubation for 4 hours in synthetic solvents.*

9.2 Stability with and without neutralization at 90°C

Resin bound PMO was found to be unstable when detritylated but not neutralized (i.e. with protonated 3' morpholine). Two samples of 10-mer PMO with the sequence 5'-CTCCAACATC-Trityl-3' were detritylated under batch conditions (**Supplementary Section 3.2.2**). One sample was subsequently neutralized before both samples were incubated at 90°C in DMI. Samples were cleaved and analyzed using LC-MS **Supplementary Section 1.2 Condition 1**. When neutralized, the resin bound PMO was stable (Supplementary Figure 9), showing no degradation peaks by LC-MS. Conversely, the sample without neutralization showed significant degradation.

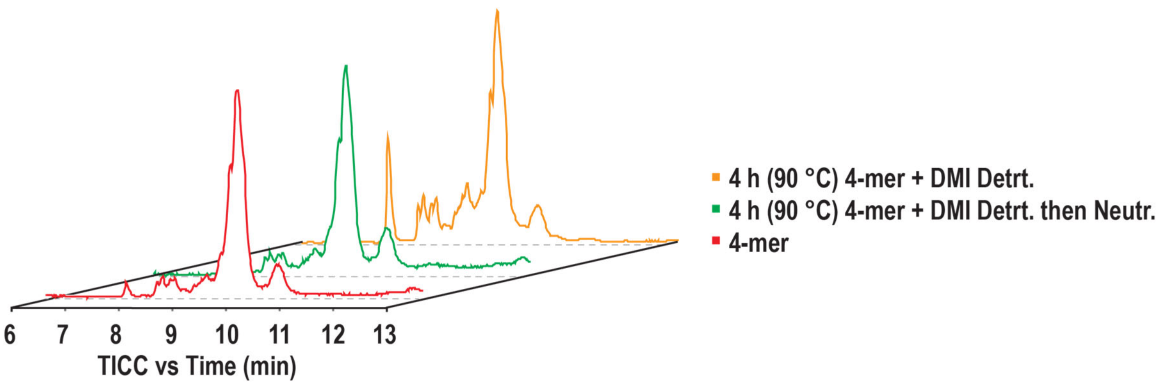

Supplementary Figure 9: Resin bound PMO is stable to high temperatures when neutralized after detritylation. *Resin bound 10-mer PMO was detritylated under batch conditions (**Supplementary Section 3.2.2**) and either neutralized or not neutralized. Samples were incubated with DMI at 90°C for 4 hours and subsequently cleaved and analyzed by LC-MS. All traces taken using Supplementary **Section 1.2 Condition 1.***

9.3 Stability to incubation in DCE at 70°C and 90°C

Samples of 10-mer PMO with the sequence 5'-CTCCAACATC-Trityl-3' were incubated in DCE at both 70 ˚C and 90 ˚C. Samples were cleaved and analyzed using LC-MS **Supplementary Section 1.2 Condition 1**, and the TICCs are shown in Supplementary Figure 10. Small amounts of the PMO was detritylated during the LC-MS separation, and eluted at 11.5 minutes. The PMO sequence shows minor degradation products after incubation at 70 ˚C for 1 hour. The side-product profile is complex, and enough is present to prevent the use of DCE for PMO synthesis at 70˚C or higher temperatures. Degradation rate increases as temperature is increased to 90 ˚C and the incubation time is increased to 4 hours.

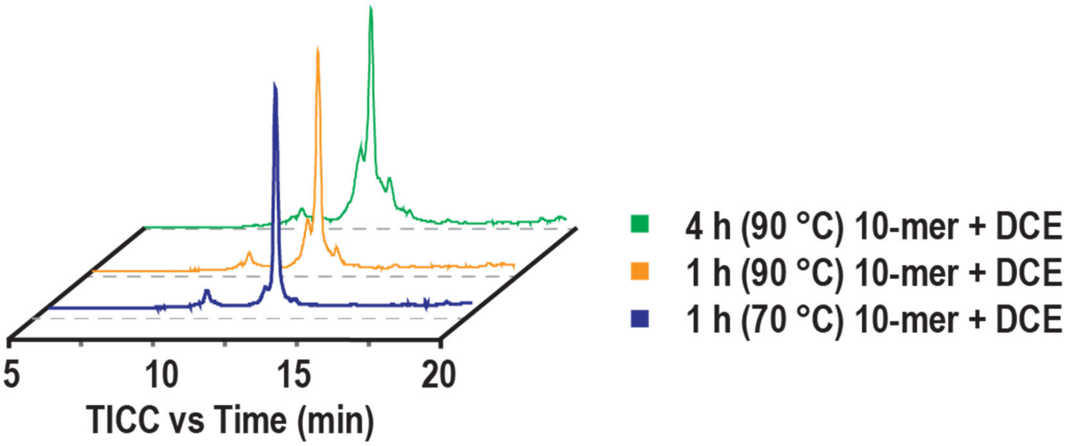

Supplementary Figure 10: Resin bound 10-mer PMO is not stable to either 70 °C or 90 °C incubation in DCE. *DCE is not a suitable solvent for PMO synthesis above room temperature. Although DCE led to better synthetic outcomes at high temperature for short sequences (see*

***Supplementary Section 7)**, resin bound PMO is not stable to incubation in this solvent at either 70 °C or 90 °C. Close eluting uncharacterized degradation products accumulated as both temperature and incubation time increased.*

9.4 Stability to batch detritylation conditions at 70 °C and 90 °C

Samples of 10-mer PMO with the sequence 5'-CTCCAACATC-Trityl-3' were detritylated under batch conditions (**Supplementary Section 3.2.2**) at 70 °C and 90 °C. Samples were cleaved and analyzed using LC-MS **Supplementary Section 1.2 Condition 1**, and the TICCs are shown in Supplementary Figure 11. The PMO showed no degradation after detritylation at 70 °C, but did show degradation after detritylation at 90 °C, especially after 4 hours. The principle degradation products were deamidation of the phosphorodiamidate linkers.

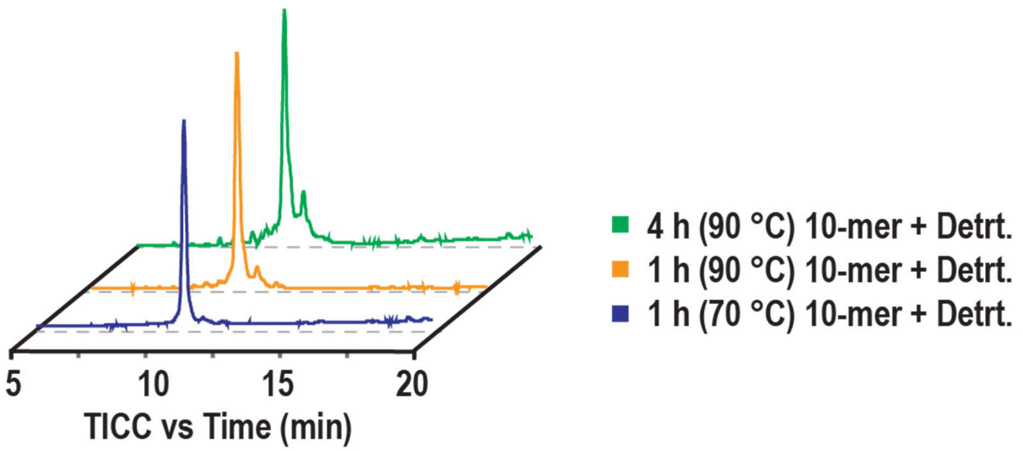

***Supplementary Figure 11: Resin bound 10-mer PMO is not stable to 90 °C incubation in batch detritylation solution.** Resin bound 10-mer PMO with the sequence 5'-CTCCAACATC-Trityl-3' was detritylated under batch conditions at high temperature. Resin was cleaved using **Supplementary Section 4 Method 2.** No degradation was observed after 1 hour at 70 °C, but incubation at 90 °C led to accumulation of side products, especially after 4 hours.*

9.5 Detritylation Optimization at 90 °C

The protected PMO chain is known to be unstable to acidic conditions[6], so use of a milder acid is required for 90 °C detritylation. Although use of typical acid mixtures at 90 °C removes the 3'-trityl group rapidly, it also severely degrades the PMO chain on the same time scale (Supplementary Figure 12). Room temperature PMO syntheses use the conjugate acid of substituted heterocycles to fine-tune acid strength[6], and we reasoned that the same strategy can be applied to find a reagent for use at 90 °C. Of special interest was the substituted pyridine class of reagents, and among these 4-cyanopyridine trifluoroacetate is the most effective in room temperature syntheses[6].

A series of less acidic substituted pyridine trifluoroacetates were tested, and 3,5-lutidine was found to lead to rapid deprotection but slow degradation (Supplementary Figure S12). A protected, resin-bound 4-mer PMO with the sequence 5'-ACGT-Trityl-3' was treated with trifluoroacetate salts of 4-cyanopyridine (control, pK_a = 1.9)[7], pyridine (pK_a = 5.2)[8], 3,5-lutidine (pK_a = 6.1)[9], and 2,4,6-collidine (pK_a = 7.5)[7] at room temperature or 90 °C. Reagent solutions include 100 mM of each pyridine trifluoroacetate in 30% trifluoroethanol (TFE), 69% DCM and 1% ethanol (v/v/v). Time points were taken at 1, 30, and 120 minutes and analyzed by LC-MS. Treatment at 120 minutes is representative of the cumulative deprotection time after a full synthesis. All reagents except collidine trifluoroacetate were able to completely remove the trityl group in one minute as tracked by the loss of trityl-on peak at 15.5 minutes in the LC-MS trace. Pyridine and cyanopyridine trifluoroacetates were too acidic and lead to degradation of the PMO chains through deamidation of the phosphorodiamidate linkages presenting at 11.9 minutes in the LC-MS trace. 3,5-Lutidine was chosen for further studies as it avoided degradation at the longest treatment time, but also led to full deprotection in under one minute.

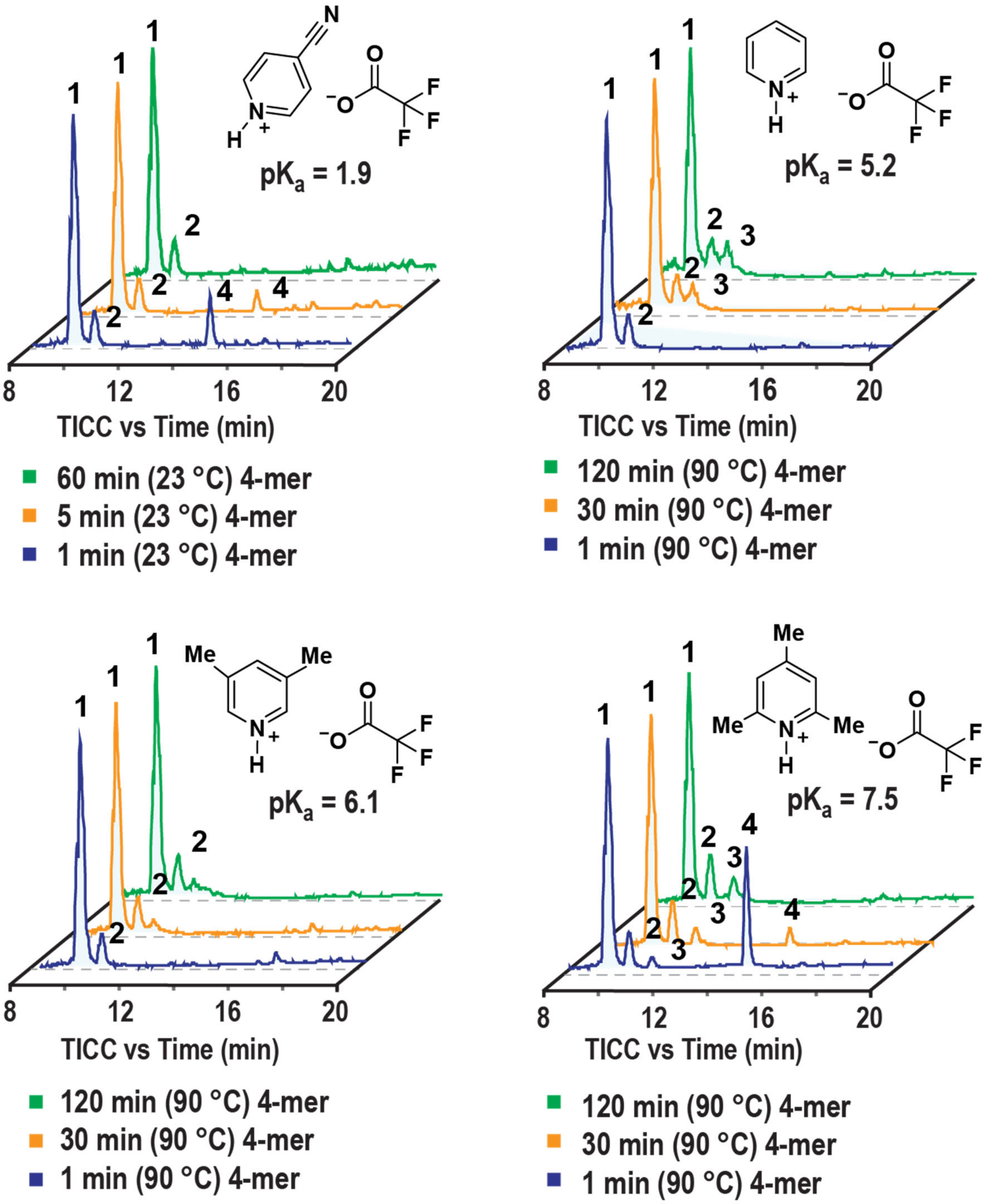

Supplementary Figure 12: *The 3'-triphenylmethyl group (Trityl) of the 4-mer PMO sequence 5'-ACGT-Trityl-3' is quantitatively removed in 10 minutes by both 4-cyanopyridine trifluoroacetate at room temperature and 3,5-lutidine at 90 °C. Trityl group loss is monitored by disappearance of peak 4, and degradation is tracked by increases in peak 3. Peaks 1 and 2 represent resolved diastereomers of the intact deprotected PMO chain.*

10. Design of a fully automated flow synthesizer

10.1 General introduction of Tiny Tides

The automated synthesizer Tiny Tides is shown in Supplementary Figure 13.

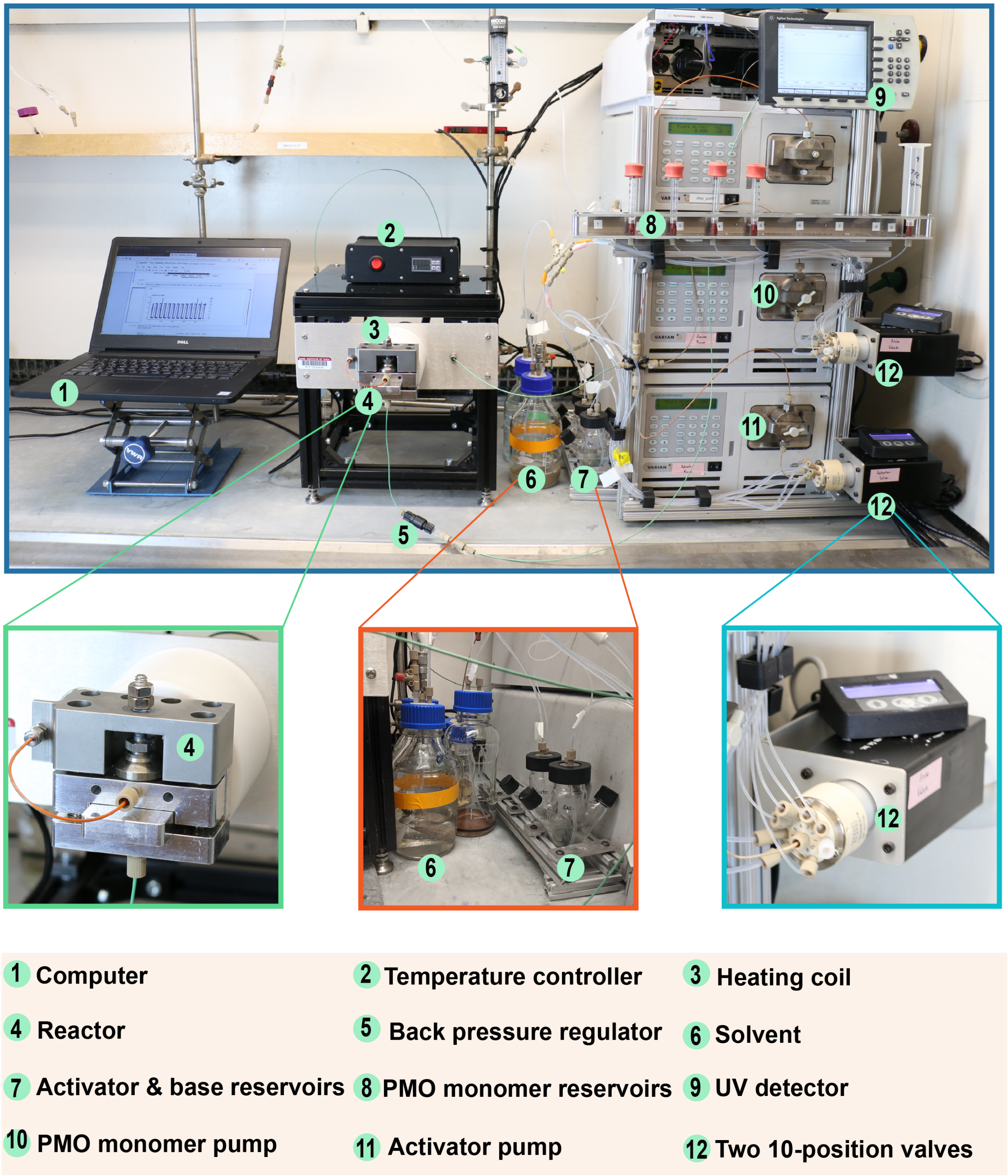

Supplementary Figure 13. Tiny Tides is a complex instrument composed of many individual parts. *Major parts are indicated with numerals, and a brief description is below. Only two HPLC pumps are labeled – parts 10 and 11 – the third was not used in this work.*

(1) Computer – The instrument is controlled with an in-house Python script[10]. The programming environment is available on an open-source GitHub repository.
(2) Temperature Controller – A Misumi temperature controller (Part #: MTMNRD) was used to drive power for an OMEGA 120V 300W heater cartridge (Part#: HDC00059).
(3) Heating Coil – 3 feet of 316L stainless steel tubing (0.064” OD x 0.020” ID) wrapped around a heated aluminum core.
(4) Reactor – Custom aluminum and stainless steel reactor head. A screw-handle on top holds a cartridge containing resin in the solvent flow-path. See **Supplementary Section 10.2** for a detailed description.
(5) Back pressure regulator – 100 PSI IDEX back pressure regulator (Part #: P-607).
(6) Solvent bottles – DMI and DCM were stored over activated 3 Å molecular sieves in two 500 mL Schott brand media bottles. The bottles were fitted with machined polypropylene caps to supply nitrogen and maintain positive nitrogen head pressure. See **Supplementary Section 10.3** for more details.
(7) Reagent reservoirs – Deprotection, neutralization, and coupling base solutions were stored in 3-necked 100 mL stirrer flasks from Chem Glass (Product #: CLS-1401-100). Top caps were custom machined from polypropylene to enable maintenance of positive pressure of dry nitrogen. See **Supplementary Section 10.3** for more details.
(8) Monomer reservoirs – Monomer solutions were stored in 5 mL polypropylene syringes under positive pressure of dry nitrogen supplied through custom polypropylene caps.
(9) UV Detector – Agilent 1260 UV detector (Part #: 1260DAD) fitted with a micro flow cell 6 mm path length (Part #:01200-90130 Rev. B). Effluent was monitored at 280 nm.
(10) Monomer HPLC Pump – Agilent 210 series HPLC pump fitted with a 5 mL SS pump head. One stroke delivers 40 μL of solution.
(11) Activator HPLC Pump – Agilent 210 series HPLC pump fitted with a 5 mL SS pump head. One stroke delivers 40 μL of solution.
(12) 10-Position valves – VICI brand EUHA body fitted with a 10-position Chem-Inert model 16P-0713L valve face.
Tubing: Tubing from the pump to the mixer T is PEEK 1/16” OD x 0.02” ID tubing (McMaster Part#: 51085K45). Tubing from the Reagents to the valves is PFA Nat 1/16” OD x 0.030” ID tubing (IDEX Part #: 1514L). All other tubing is PEEK 1/16” OD x 0.030” ID tubing (McMaster Part#: 51085K48).

Fittings: Unless otherwise specified, all fittings were IDEX brand Super Flangeless™ PEEK fittings.

10.2 Reactor design

The reactor chamber that holds the solid support is an important factor in flow instruments with solid supported reactants[11]. Geometry, temperature flux, and reactor dead volume are significant factors that can affect reaction efficiency and economy[12]. In this work, we designed a reactor that scaled down the optimized geometry previously reported for flow peptide synthesizers that utilize a similar solid support for solid phase peptide synthesis [13]. The reactor used in our instrument, dubbed Tiny Tides, maintains the reported geometric ratios, but is scaled down by a factor of 10 to accommodate a 4.5 micromole synthesis (~10 mg resin in ~600 μL). This smaller reactor saved expensive reagents, while still producing enough PMO for initial biological studies.

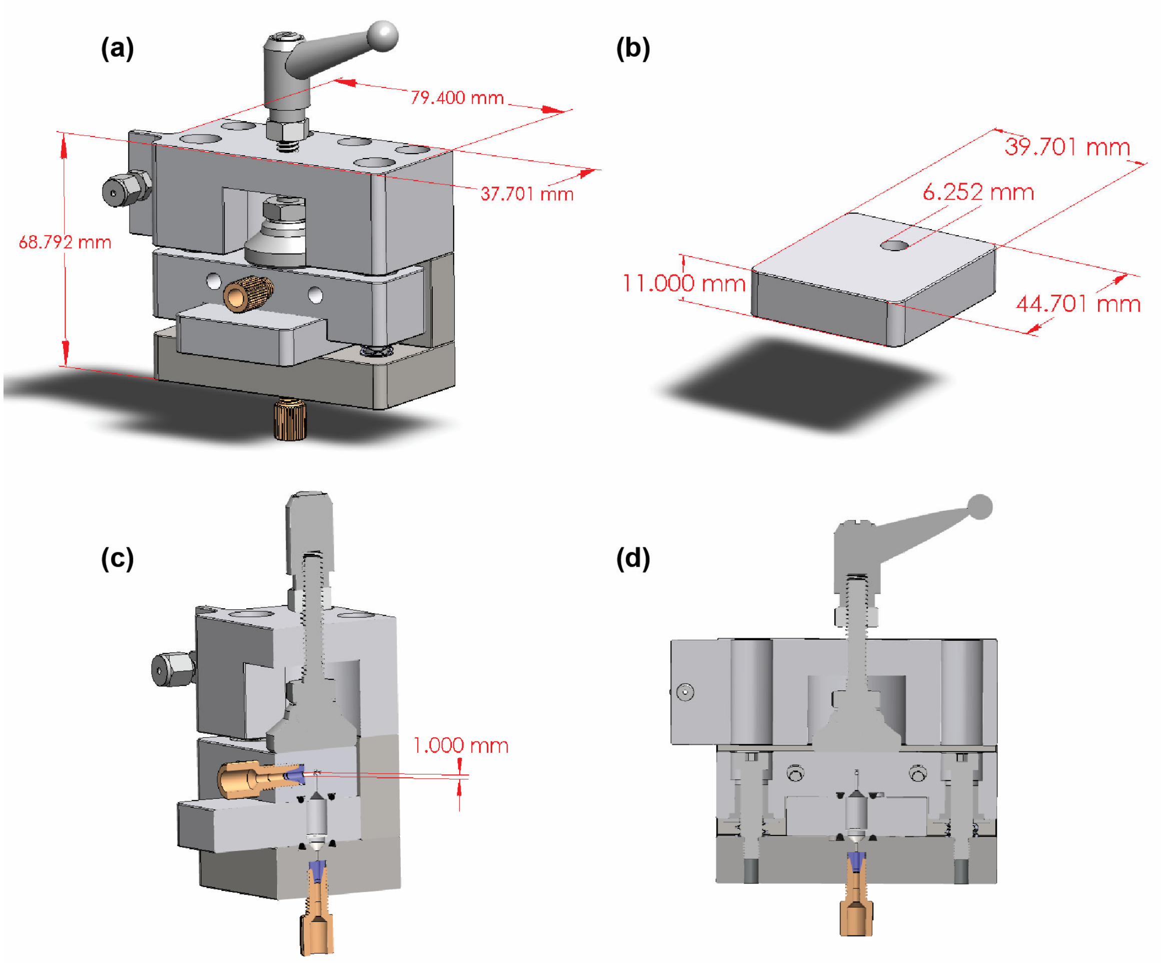

***Supplementary Figure 14. Computer Aided Design (CAD) Projection of the optimized Reactor Design**. a) The reactor design is shown along with the cassette used to hold the resin. b) The inner cartridge that contains the resin is shown. Resin is retained in the thru-hole. c) Cross-section view of the reactor is shown, revealing the inner resin chamber. Flow enters through the PEEK fitting in the centre, passes through the cartridge hole, and exits through the fitting in the bottom. The cartridge is held in place with the screw clamp from above. d) A cross-section view of the reactor from the front face is shown.*

To ensure rapid turnover between syntheses and minimize maintenance down-time, a quick-release system was implemented to manage resin holders. Most importantly, the reactor must be stable to operation at high enough pressures to allow for the use of dichloromethane at 90 °C where its vapor pressure is about 5 bar. A cartridge-based system held resin in a reusable metal container that could be placed in the flow path and clamped down to withstand expected pressures. All pieces were initially machined from aluminum to ensure efficient heat transfer. A final revision of the reactor body changed the top half of the reactor to stainless steel to allow for larger clamping pressures (Supplementary Figure 14).

10.3 Positive nitrogen pressure

The monomer solutions are sensitive to moisture, so we kept them under anhydrous nitrogen pressure. The slight positive pressure also helped eliminate cavitation in the pump heads. The plumbing system for dry nitrogen is shown in Supplementary Figure 15.

The containers used for monomer storage include an exit path to allow for continuous nitrogen flow, and flow to the monomers was controlled with an in-line air flow controller. Continuous nitrogen flow helps remove trace water that infiltrates the system of remains in the DMI used for monomer stock solution. This precaution is not required for the other reagents because they are less sensitive to moisture. The excess nitrogen flow from the intentionally leaky reagent containers would consume a large majority of the nitrogen flow if not regulated with the upstream flow limiter.

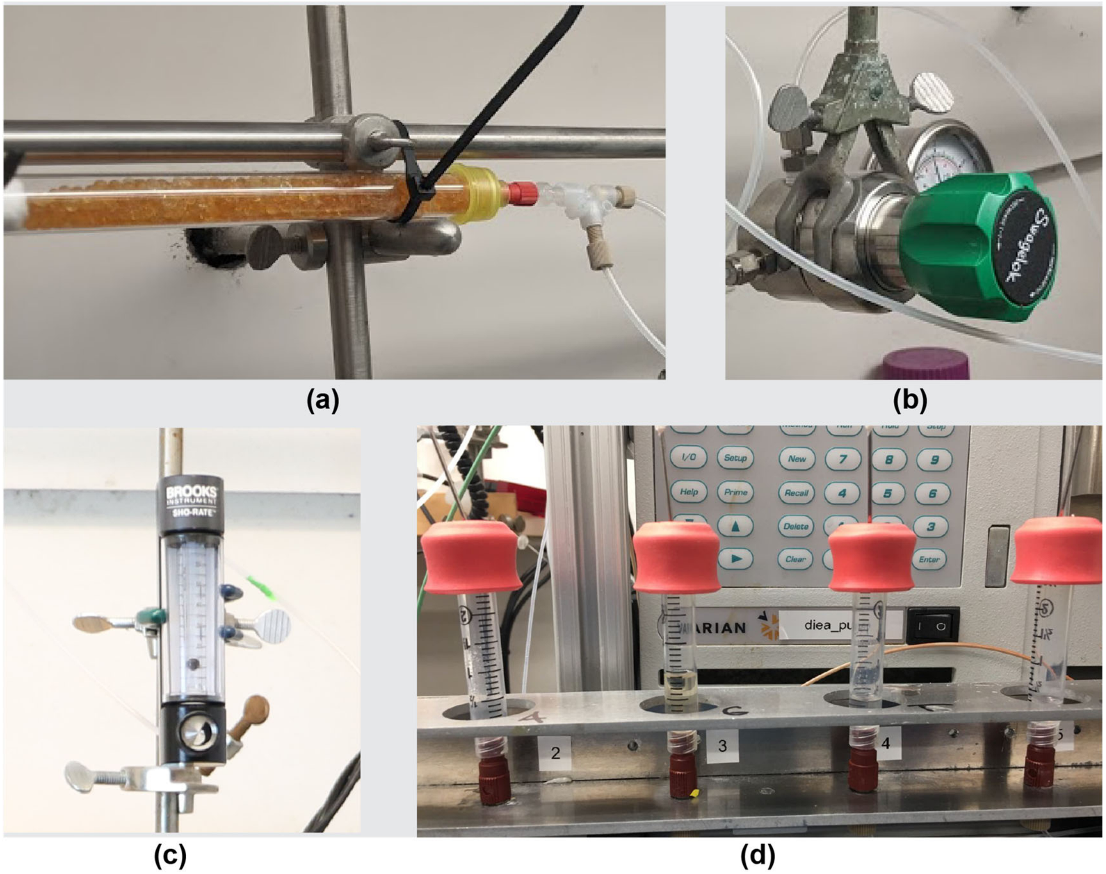

Supplementary Figure 15. Nitrogen plumbing on the Tiny Tides allows for anhydrous storage of activated monomer solutions. *a) Nitrogen from the hood manifold is plumbed through an activated silica gel desiccant column. b) An in-line pressure regulator allows for precise back pressure control over the entire system. c) A nitrogen flow limiter is placed before the monomer solutions but not the reagent lines to prevent excess nitrogen leakage from the monomer containers. d) Monomer solutions are stored in disposable syringes with nitrogen pressure plumbed in through the top.*

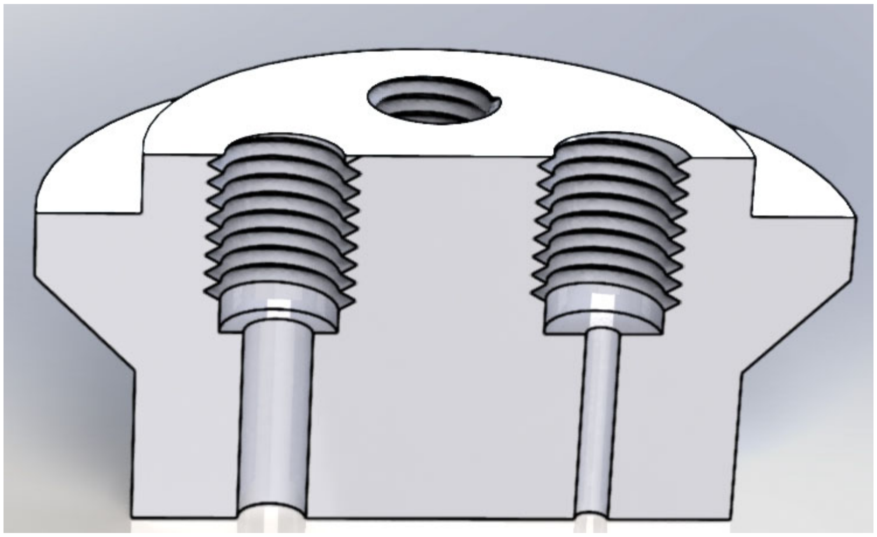

Supplementary Figure 16. Reagent bottles are kept under positive nitrogen pressure with a machined adapter. *Schematic of the cap adapters that were machined to provide connection points for solution and nitrogen lines.*

Other reagents were held in glass containers under 5 PSI of nitrogen without continuous nitrogen flow. Nitrogen was supplied to GL-45 threaded bottles through a custom-machined polypropylene adapter for use with GL-45 caps with holes (Chemglass). These adaptors had three ¼-28 threaded ports. Two ports had 1/8" thru holes; the third had a 1/16" thru hole. The first hole was used for tubing to deliver reagent to the valves. The second contained a Swagelok® miniature Quick-Connect fitting for the anhydrous transfer of solvents, and the third was used for the nitrogen gas supply. The line used for reagent transfer was inserted into the bottom of the reagent reservoir and sealed in place with a Super Flangeless™ fitting (Idex XP-131). Nitrogen gas was supplied by a 1/8" line seated against the 1/16" thru hole and sealed in with a Super Flangeless™ fitting. The fill port consisted of a thin wall 1/8" OD, 0.1" ID, stainless steel tube inserted to just below the bottom of the machined adaptor and sealed in place with a Super Flangeless™ fitting. The free side was fitted with a shut off Quick-Connect fitting (Swagelok SS-QM2-B-200KR) that was sealed when not in use but could be used to fill the reservoir from a second reservoir of anhydrous solvent under slightly higher pressure. A schematic showing the polypropylene bottle-top adapter is shown in Supplementary Figure 16.

10.4 Mixer design

Effective mixing of the streams from the two pumps is critical for effective PMO synthesis. Although there exists extensive research into more effective mixing Ts[14], we found that use of a simple T union (Idex part #P-722) was

adequate. Pump streams entered opposite each other at high flow leading to sufficient mixing for PMO synthesis (Supplementary Figure 17).

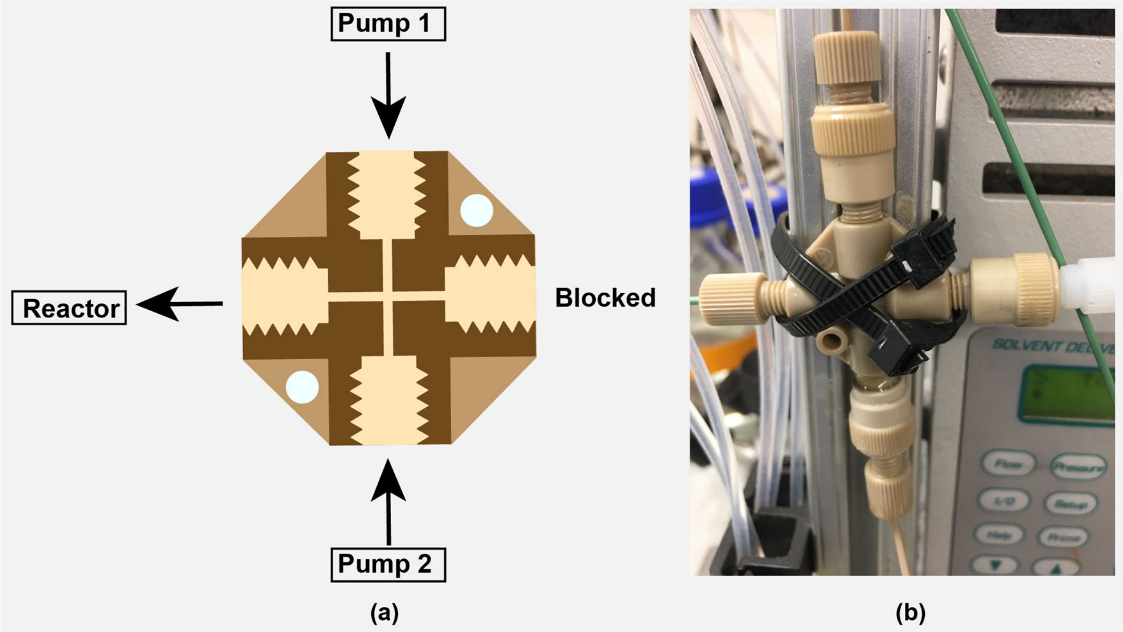

Supplementary Figure 17. Mixing of the two pump flows is accomplished using a T mixer. *a) The inner schematic for the T mixer used is shown. Flow from the two pumps pass through check valves and meet opposite each-other and exit through another channel. The remaining channel is not used and is blocked during synthesis. b) The streams from the two pumps meet from opposite directions in a PEEK cross-mixer. The arm shown on the right is not used for PMO synthesis.*

10.5 UV-Vis monitoring

An in-line UV-Vis monitor installed after the reactor was used to monitor the delivery of each reagent. Although the bright yellow trityl cation released during deprotection is an attractive way to monitor reaction progress, chemicals are included in the optimized detritylation reagent rapidly quench the trityl cation and prevent it from reacting with the resin bound PMO. The quenched species have overlapping UV absorbance with the deprotection regent, restricting use of the UV detector to monitoring mechanical function of the instrument in routine operation. To rapidly optimize reaction parameters, deprotection mixtures without trityl cation quenchers were used to track the efficiency of PMO synthesis in real time. A modified synthesis protocol was developed to use 3% trichloroacetic acid in DCM (w/v) for deprotections. Although the resulting PMO showed significant impurities from acidolysis similar to those described in **Supplementary Section 9.4**, monitoring trityl removal after coupling enabled rapid optimization of the coupling step in real time without cleavage and LCMS analysis.

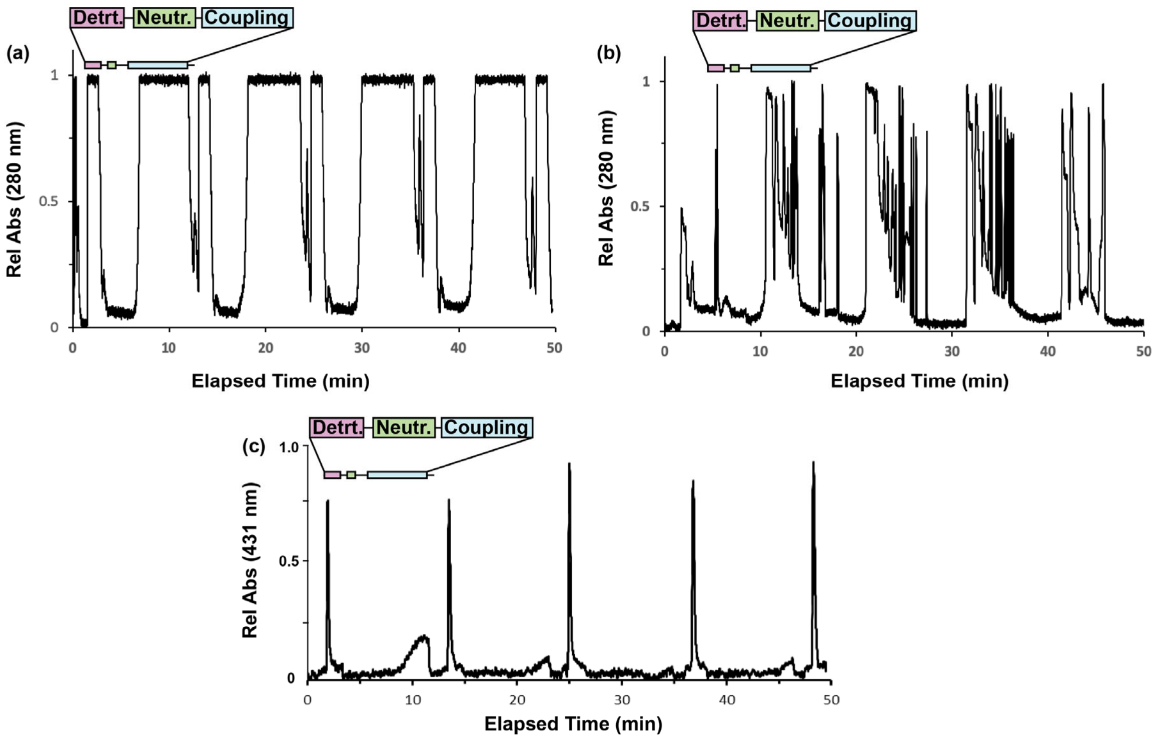

Supplementary Figure 18. In-line UV-Vis monitoring is a powerful tool for tracking instrument performance*. a) Reagent delivery during an efficient synthesis is consistent, as monitored by UV-Vis absorbance at 280 nm. b) System leaks cause a distinct, noisy UV-Vis trace. c) Use of a spectroscopically-silent deprotection mixture allows for tracking real-time reaction efficiency via monitoring trityl cation release at 431 nm.*

In normal operation, however, We used the in-line UV-Vis detector to track real time instrument performance and ensure proper pump coordination, valve timings, software triggers, and other key parameters. A typical reaction trace along with one associated with improper reagent delivery is shown in Supplementary Figure 18. The details of the traces were used to identify which part of the instrument was malfunctioning, greatly reducing repair and optimization times.

10.6 Pump head cavitation

Unoptimized pump delivery cycles led to consistent under delivery of the PMO monomer solutions, with the default configuration delivering only 2/3 of the expected volume. Although there are many possible reasons for this behavior, including mechanical restrictions in the valve face, incorrect valve timings, or other software bugs, it only affected monomer solutions and some were more affected than others. This evidence suggested that the problem was caused by the composition of the monomer solutions.

We ultimately determined that cavitation in the pump head was responsible for the observed under-delivery, with under-delivery proportional to the viscosity of the solution. Monomer solutions are very concentrated (0.8 M) and as a result are very viscous. This problem is especially bad for moG, and less pronounced for moT, likely due to the much higher molecular weight of moG.

The Varian 210 HPLC pumps in the instrument are single piston reciprocating positive displacement pumps specifically selected for their very low non-displaced volume. The low non-displaced volume enables changeover of solvents and reagents in very few pump strokes (99% change in composition in about 3 strokes). The drawback of this pump design, however, is that the flow is discontinuous. There is a discrete delivery phase where the piston compresses fluid within the pump head and ejects it through the outlet, and a discrete withdraw phase where the piston withdraws and sucks fluid into the pump head. During the delivery phase, no new fluid is entering the pump head and during the withdraw phase there is zero outlet flow rate (Supplementary Figure 19a). This means that the pump must balance the withdraw and delivery rates to achieve the selected total flow rate.

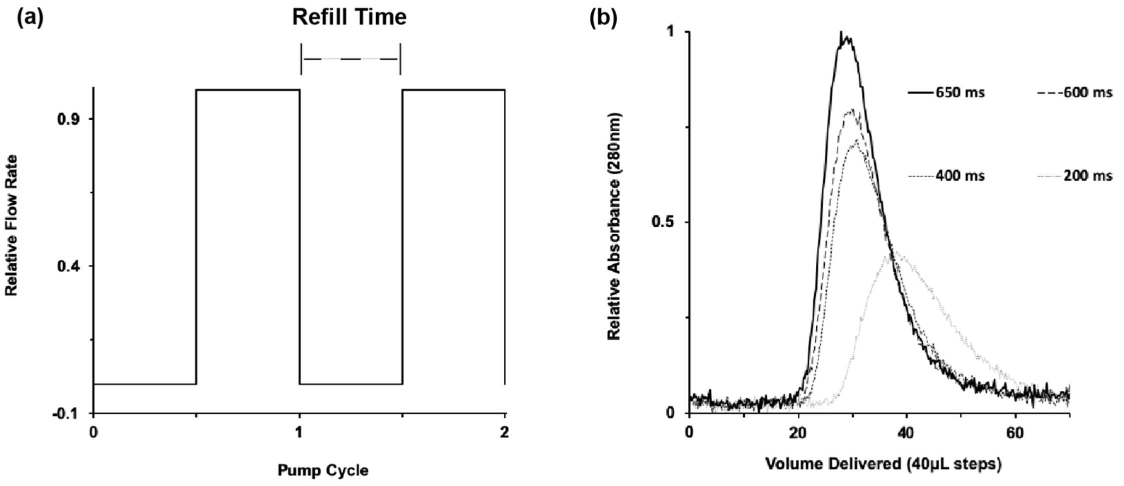

Supplementary Figure 19. Pump head cavitation can be alleviated with long refill times. *a) Schematic showing the flow rate as a function of time for the HPLC pumps used to deliver reagents. Increasing refill time results in higher relative flow rates with longer gaps in-between. b) Increasing the refill time results in better delivery of viscous reagent mixtures.*

By default, the withdraw stroke is rapid to minimize discontinuities in the flow rate when pumping low viscosity solvents like water and acetonitrile. When connected to high viscosity solvents, however, there is no time for the viscous fluid to fill the pump head during the withdraw stroke. The piston will withdraw, creating a reasonable vacuum (~29"Hg), but the fluid cannot creep into the chamber fast enough. As a result, the delivery stroke collapses vacuum for part of its stroke,

then delivers as normal. The result is a short observed delivery stroke that delivers less than the expected volume.

To confirm cavitation was the problem, the nitrogen head pressure on the monomers was increased. This fixed the cavitation and under-delivery of monomer, but was mechanically inconvenient to maintain. Instead, the pump withdraw time was increased from the default 200 ms to 650 ms to give the solution more time to fill the chamber on each stroke. To optimize the pump withdraw time, delivery of concentrated food dye in DMI was pumped, and the relative delivery of the dye solution was quantified by tracking with the in-line UV monitor (Supplementary Figure 19b). As refill time was increased, increasing amounts of monomer solution were delivered to the UV monitor. For all further studies, we chose the maximum refill time that the pumps can use when delivering 2.5 mL/min, 650 ms. It is likely that cavitation is still present in the system at this setting, but further increases in refill time would limit the maximum flow rate. We determined that the cavitation at this setting is likely not significant, as the total consumed volume of the most viscous monomer solution, moG, after 5 consecutive couplings delivers 98% of the total theoretical volume (50 μL remaining of 1.6 mL to be delivered)

11. PMO chain stability under optimized conditions

The synthetic conditions obtained from the 4-mer and instrument optimization studies are different from the on-resin stability studies in **Supplementary Section 9,** so stability studies were repeated. A protected resin-bound PMO with the sequence 5'-ACGT-Trityl-3' was incubated at 90 °C in the optimized reagent solutions listed in **Supplementary Section 2.2**, diluted to the final in-reactor composition as appropriate. Sample resins were cleaved using **Supplementary Section 4 method 1** and analyzed using **LCMS method 1.2 condition 2**. Results are shown in Supplementary Figure 20. The PMO does not degrade in any of the tested conditions. Thermal degradation of the PMO sequence is characterized by many closely-eluting side products and is not observed in these experiments. The late eluting peak at 12.5 minutes in the LC-MS separation corresponds to the PMO sequence that retains the 3' trityl group, as trityl groups are partially lost in the LC-MS method leading to the production of two peaks. The two major peaks are not associated with degradation under the incubation conditions.

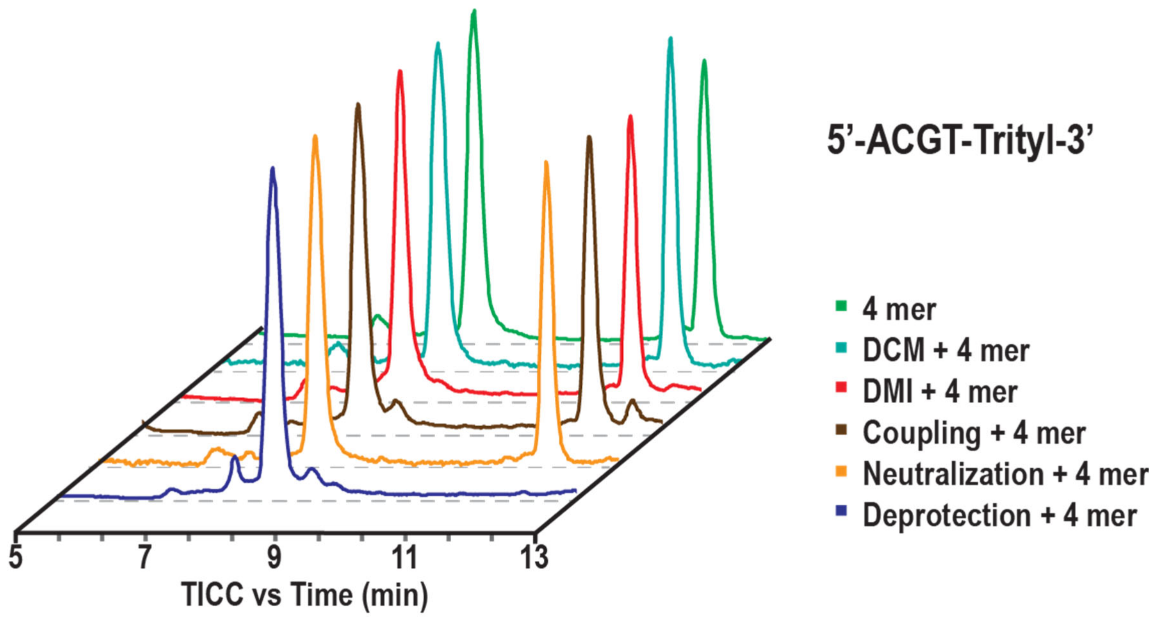

Supplementary Figure 20. Resin bound 4-mer PMO is stable to optimized synthesis conditions at 90 °C. *Protected PMO with the sequence 5'-ACGT-Trityl-3' still bound to resin was incubated in the optimized reagent solutions from **Supplementary Section 9** diluted to the final in-reactor compositions as appropriate. Degradation was tracked using LC-MS, and the closely-eluting side product peaks characteristic of degradation products were not observed. Both major peaks at 8.5 minutes and 12.5 minutes in the LC-MS separation correspond to the intact PMO sequence. The late-eluting peak retains the 3' trityl group which is partially lost under the LC-MS separation conditions.*

12. Optimized automated synthesis sequence

The synthesis order is characterized in Supplementary Figure 21. For each chain elongation cycle, steps 1-8 are repeated, and an additional deprotection sequence (steps 2-6) is run at the end of synthesis to remove the final 3' trityl protecting group. Synthetic difficulty increases as the PMO sequence gets longer, so for PMO sequences between 10 and 20 nucleotides the number of pump strokes was increased from 5 to 6, and for sequences between 21 and 30 nucleosides 7 strokes of monomer solution were used to realize a highly efficient synthesis without significantly effecting the synthetic timeline.

Step #	Description	Monomer Pump	Reagent Pump	Time	Flow Rate
**1**	Wash	DMI	DMI	90 sec	2.5 mL/min x 2 pumps
**2**	Wash	DCM	DCM	30 sec	2.5 mL/min x 2 pumps
**3**	Deprotection	DCM	***Detritylation stock***	114 sec	2.5 mL/min x 2 pumps

**4**	Wash	DCM	DCM	18 sec	2.5 mL/min x 2 pumps
**5**	Neutralization	DCM	***Neutralization stock***	12 sec	2.5 mL/min x 2 pumps
**6**	Wash	DCM	DCM	24 sec	2.5 mL/min x 2 pumps
**7**	Wash	DMI	DMI	30 sec	2.5 mL/min x 2 pumps
**8**	Coupling	***Coupling base stock***	***Monomer stock***	5 sec	2 mL/min x 2 pumps
**9**	Wash	DMI	DMI	7 sec	2 mL/min x 2 pumps
**10**	Wash	DMI	DMI	420 sec	0.1 mL/min x 2 pumps

Supplementary Figure 21. *Summary of coupling and deprotection steps performed during automated flow PMO synthesis.*

13. Raw LCMS data

13.1 LCMS traces of 4-mer PMOs for condition optimization

A series of 4-mer PMOs were produced to optimize PMO synthesis in flow (**Fig. 4**). Five mg of each resin bound PMO product was washed with DCM and dried under vacuum. After cleavage using **Supplementary Section 4 Method 1**, crude cleavage mixtures were analyzed with LC-MS using **Supplementary Section 1.2 Condition 4**. All the samples were quantified following the method described in **Supplementary Section 6**.

Note 1: Peaks outside of 6.5 - 8.0 minutes were excluded from analysis. Peaks within 6.5 - 8.0 minutes cover the desired product and structurally related side-products while compounds outside of this range are small molecules generated during the cleavage process. These peaks corresponding to small molecules from cleavage were excluded to simplify purity interpretations.

Note 2: Purity is most accurately assessed using the method described in **Supplementary Section 6**. Although TICCs and mass spectra for certain conditions may contain many products, it is not necessarily indicative of poor synthesis quality, as side-products arise during the analytical characterization itself. This class of compounds includes cytosine residues methylated during the cleavage process, incomplete removal of benzoyl protecting groups, and guanosine nucleobases eliminated during the ionization process. Relative amounts of these compounds vary greatly from sample handling and preparation,

and do not reflect changes in fast flow synthesis performance. Purity assessments made using the method described in **Supplementary Section 6** do not count these compounds as side-products.

1. Fig. 4, Entry 1
Sequence (5' to 3'): Tail-ACGT (4-mer)
Condition: Batch synthesis (**Supplementary Section 3**)
LCMS Method: **Condition 4**
Observed: 1601.63 Da Calculated: 1601.60 Da

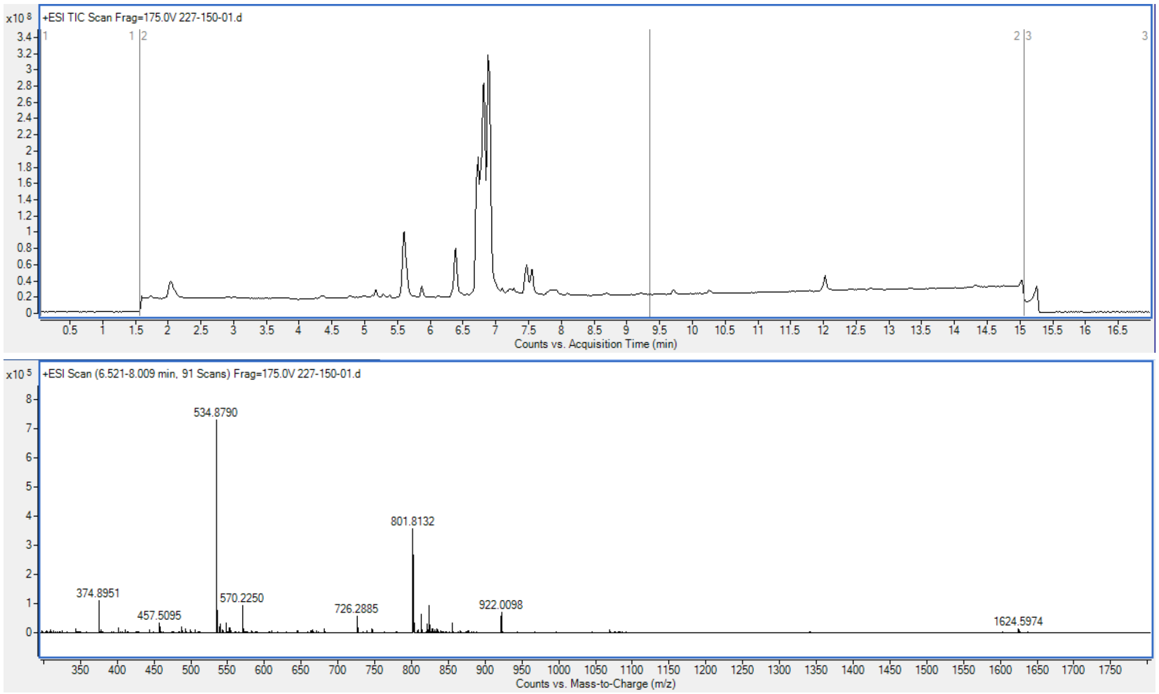

2. Fig. 4, Entry 2

Sequence (5' to 3'): Tail-ACGT (4-mer)

Condition: Tiny Tides synthesis, 70 °C, 4-cypy·TFA, DIEA neutr, DIEA base, no additive, 10 eq monomer, 17.4 min.

LCMS Method: **Condition 4**

Observed: 1601.62 Da Calculated: 1601.60 Da

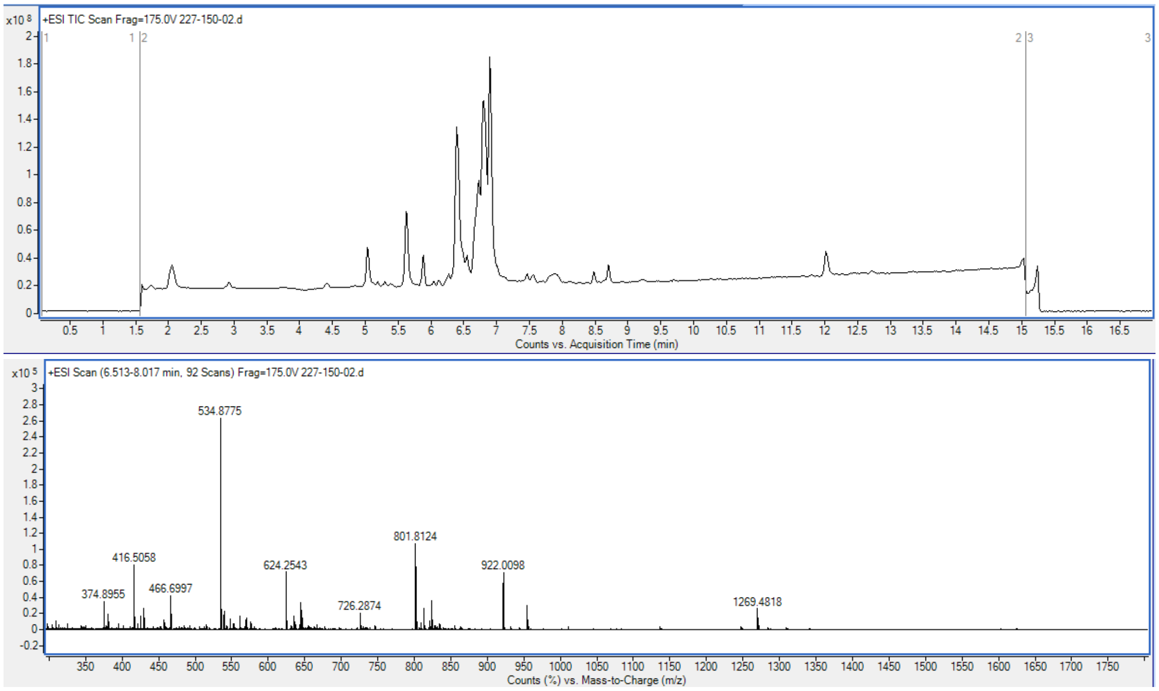

3. Fig. 4, Entry 3

Sequence (5' to 3'): Tail-ACGT (4-mer)

Condition: Tiny Tides synthesis, 70 °C, 4-cypy·TFA, DIEA neutr, DIEA base, no additive, 10 eq monomer, 11.7 min.

LCMS Method: **Condition 4**

Observed: 1601.63 Da Calculated: 1601.60 Da

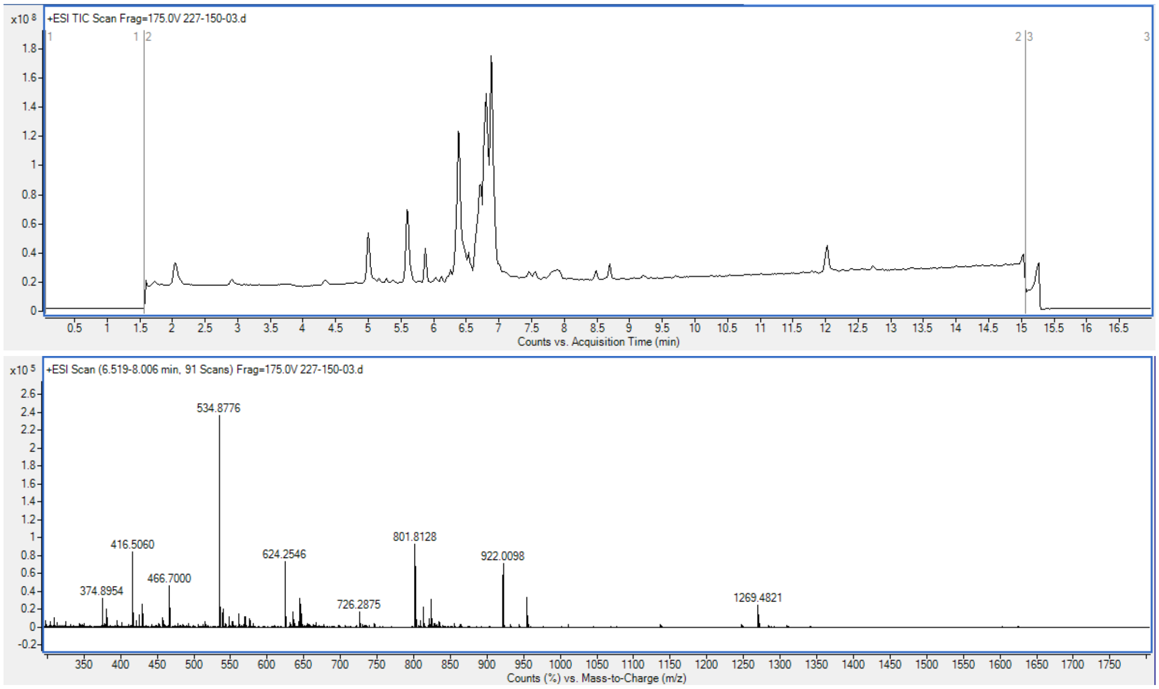

4. Fig. 4, Entry 4

Sequence (5’ to 3’): Tail-ACGT (4-mer)

Condition: Tiny Tides synthesis, 70 °C, 4-cypy·TFA, DIEA neutr, DIEA base, no additive, 18 eq monomer, 11.7 min.

LCMS Method: **Condition 4**

Observed: 1601.63 Da Calculated: 1601.60 Da

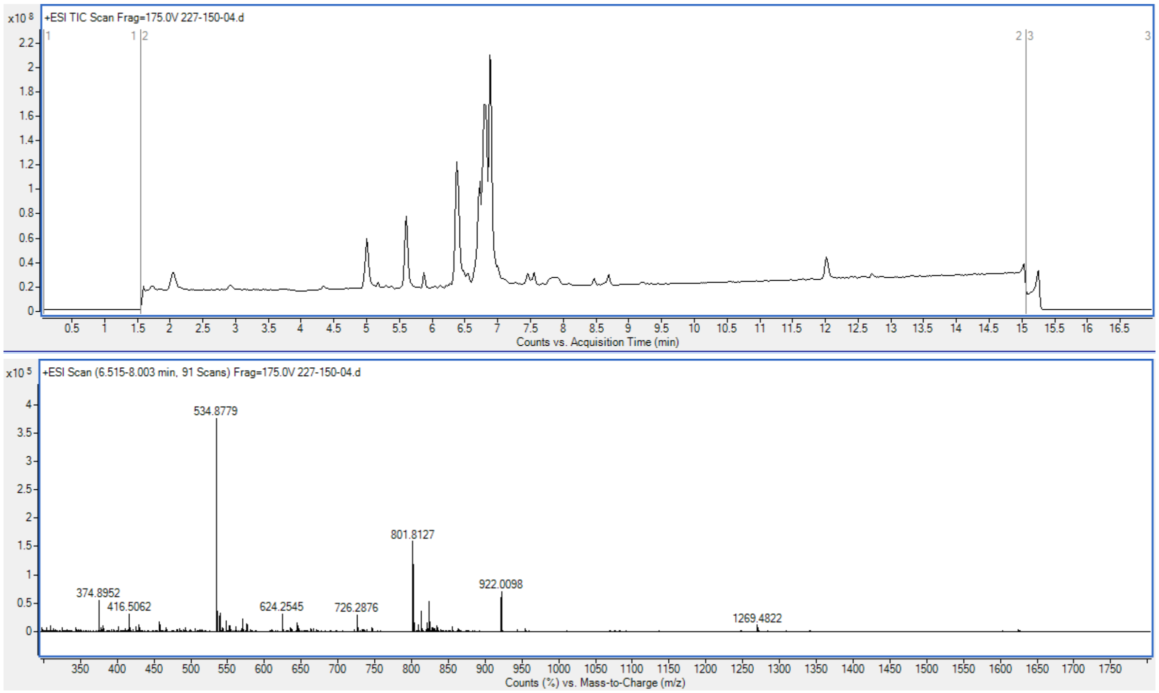

5. Fig. 4, Entry 5

Sequence (5' to 3'): Tail-ACGT (4-mer)

Condition: Tiny Tides synthesis, 70 °C, 4-cypy·TFA, DIEA neutr, DIEA base, NMI 0.2 M, 18 eq monomer, 11.7 min.

LCMS Method: **Condition 4**

Observed: 1601.63 Da Calculated: 1601.60 Da

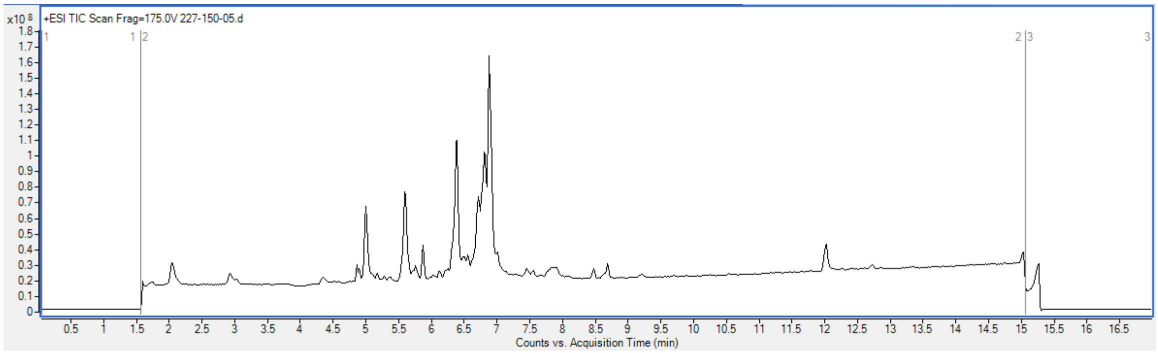

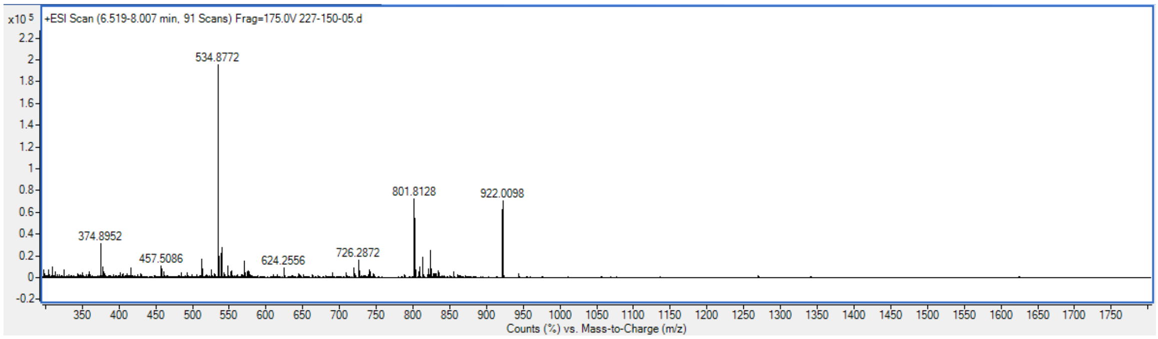

6. Fig. 4, Entry 6

Sequence (5' to 3'): Tail-ACGT (4-mer)

Condition: Tiny Tides synthesis, 70 °C, 4-cypy·TFA, DIEA neutr, DIEA base, LiBr 0.2 M, 18 eq monomer, 11.7 min.

LCMS Method: **Condition 4**

Observed: 1601.63 Da Calculated: 1601.60 Da

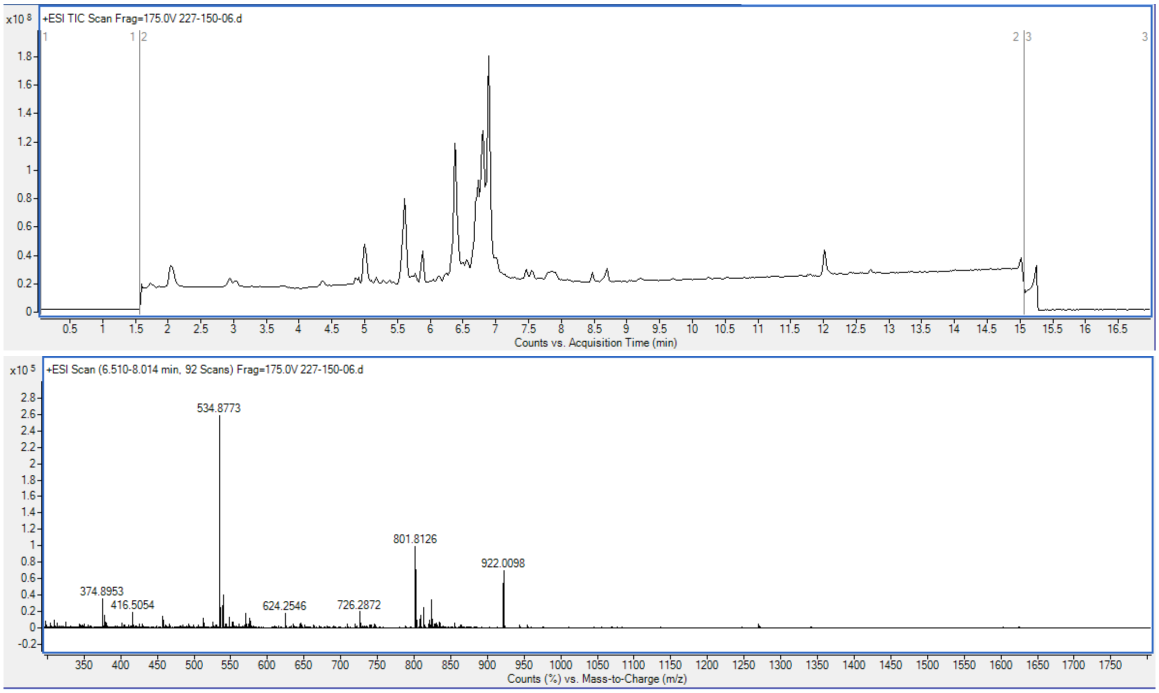

7. Fig. 4, Entry 7

Sequence (5' to 3'): Tail-ACGT (4-mer)

Condition: Tiny Tides synthesis, 90 °C, 4-cypy·TFA, DIEA neutr, DIEA base, LiBr 0.2 M, 18 eq monomer, 11.7 min.

LCMS Method: **Condition 4**

Observed: 1601.62 Da Calculated: 1601.60 Da

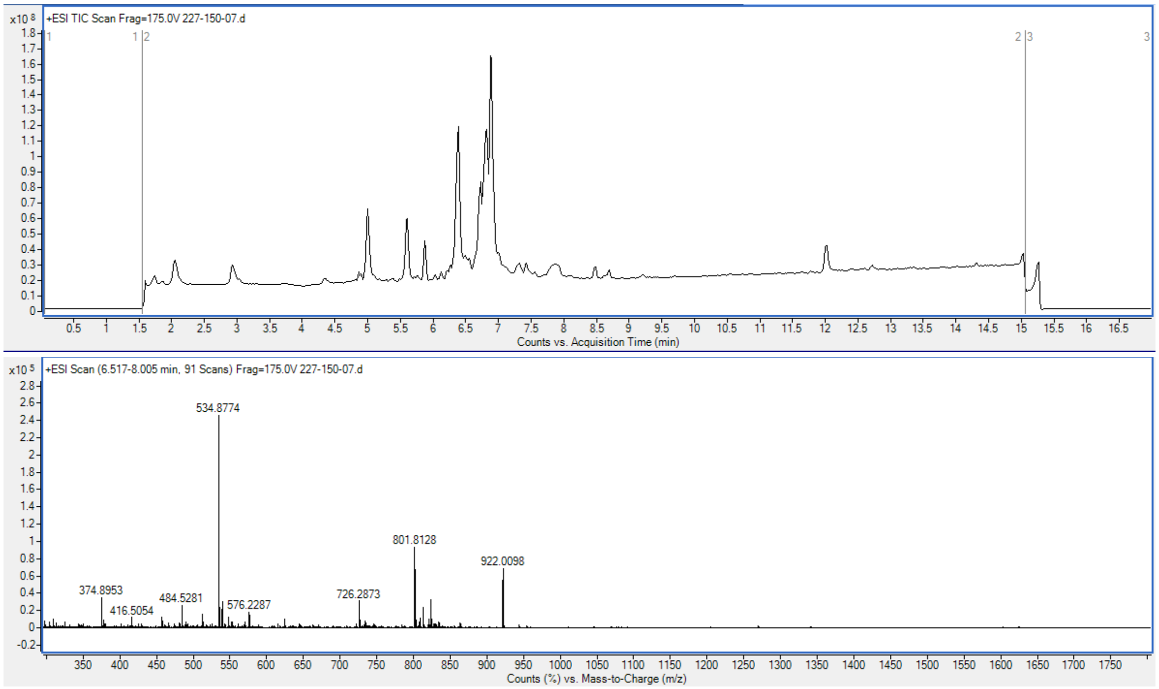

8. Fig. 4, Entry 8

Sequence (5' to 3'): Tail-ACGT (4-mer)

Condition: Tiny Tides synthesis, 90 °C, 3,5-lut·TFA, DIEA neutr, DIEA base, LiBr 0.2 M, 18 eq monomer, 12.6 min.

LCMS Method: **Condition 4**

Observed: 1601.63 Da Calculated: 1601.60 Da

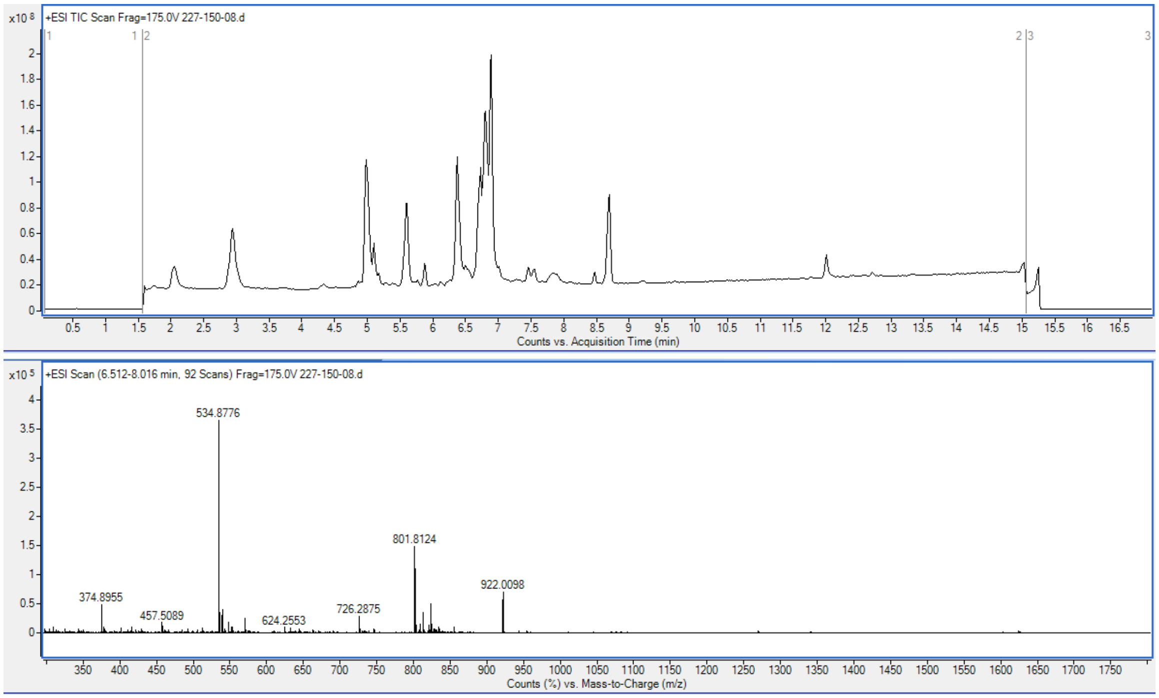

9. Fig. 4, Entry 9

Sequence (5' to 3'): Tail-ACGT (4-mer)

Condition: Tiny Tides synthesis, 90 °C, 3,5-lut·TFA, DIEA neutr, NEM base, LiBr 0.2 M, 18 eq monomer, 12.6 min.

LCMS Method: **Condition 4**

Observed: 1601.63 Da Calculated: 1601.60 Da

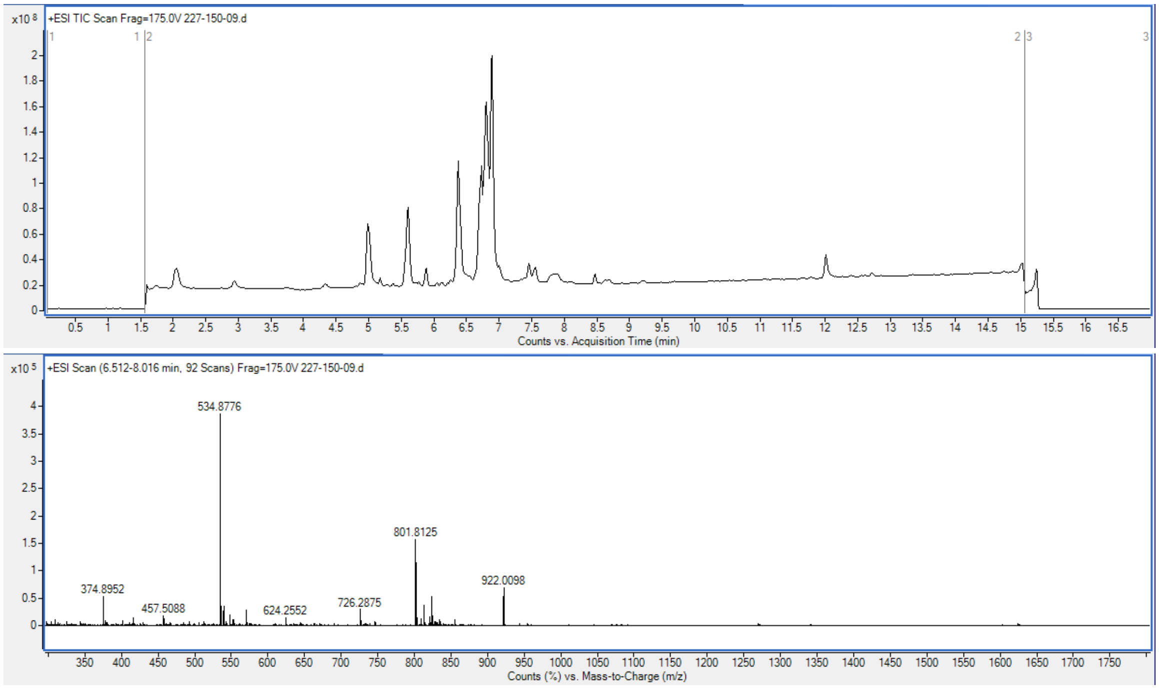

10. Fig. 4, Entry 10

Sequence (5’ to 3’): Tail-ACGT (4-mer)

Condition: Tiny Tides synthesis, 90 °C, 3,5-lut·TFA, NEM neutr, DIEA base, LiBr 0.2 M, 18 eq monomer, 12.6 min.

LCMS Method: **Condition 4**

Observed: 1601.62 Da Calculated: 1601.60 Da

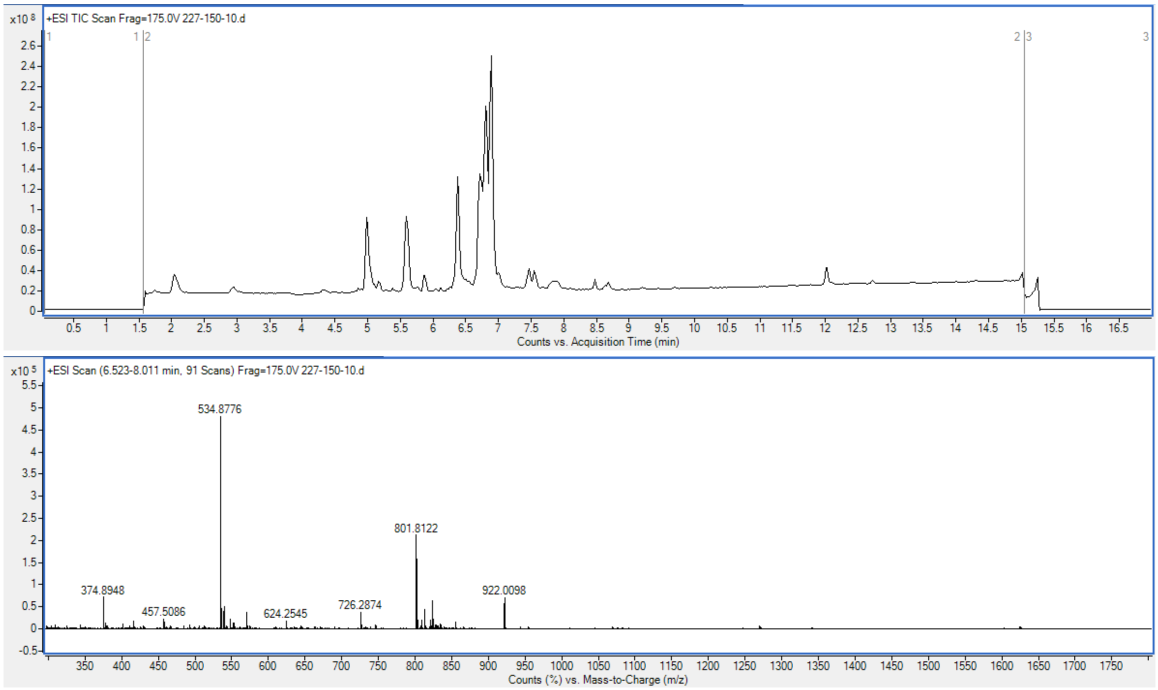

11. Fig. 4, Entry 11

Sequence (5' to 3'): Tail-ACGT (4-mer)

Condition: Tiny Tides synthesis, 90 °C, 3,5-lut·TFA, NEM neutr, DIEA base, LiBr 0.2 M, 18 eq monomer, 12.7 min.

LCMS Method: **Condition 4**

Observed: 1601.63 Da Calculated: 1601.60 Da

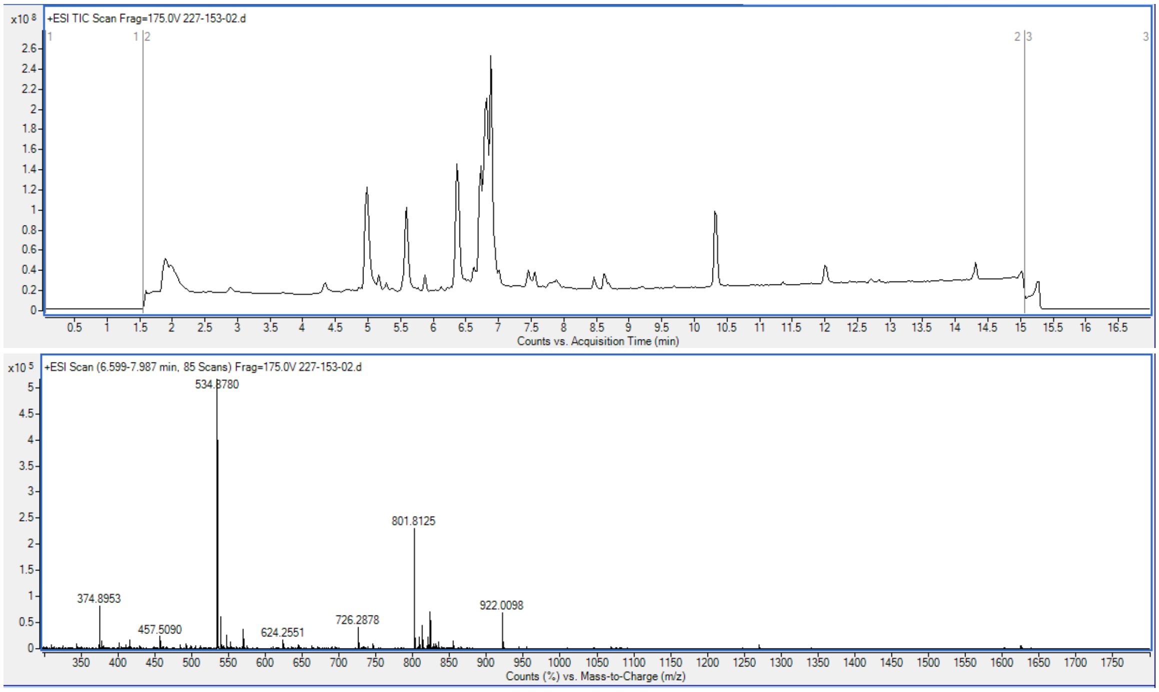

12. Fig. 4, Entry 12

Sequence (5' to 3'): Tail-ACGT (4-mer)

Condition: Tiny Tides synthesis, 90 °C, 3,5-lut·TFA, NEM neutr, DIEA base, LiBr 0.2 M, 10 eq monomer, 22.1 min.

LCMS Method: **Condition 4**

Observed: 1601.63 Da Calculated: 1601.60 Da

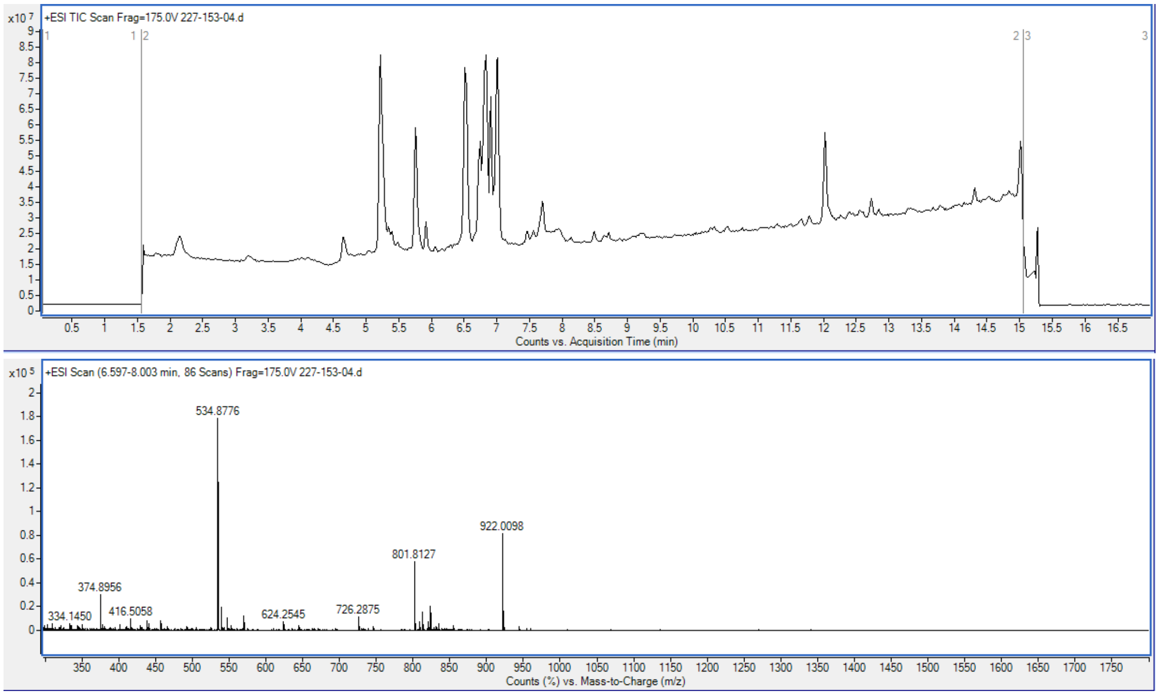

13.2 LCMS data of 12-mer with Tiny Tides 70˚C synthesis

As mentioned in Supplementary Figure 9, at 90 ˚C, 4-cyanopyridine·TFA solution will lead to acidolysis of the resin bound PMO. Herein, synthesis at 70˚C with Tiny Tides was also studied. A 12-mer PMO was successfully synthesized with Tiny Tides at 70˚C using 4-cyanopyridine·TFA solution for detritylation, DIEA and LiBr were used for coupling, NEM was used for neutralization. Cleavage was performed using **Supplementary Section 4 Method 1**.

13.2.1 Batch synthesis of 12-mer PMO

Sequence (5' to 3'): Tail-ACGTACGTACGT (12-mer)
Condition: Batch synthesis (**Supplementary Section 3**), 3-day synthesis.
LCMS Method: **Condition 1**
Observed: 4280.64 Da Calculated: 4280.52 Da

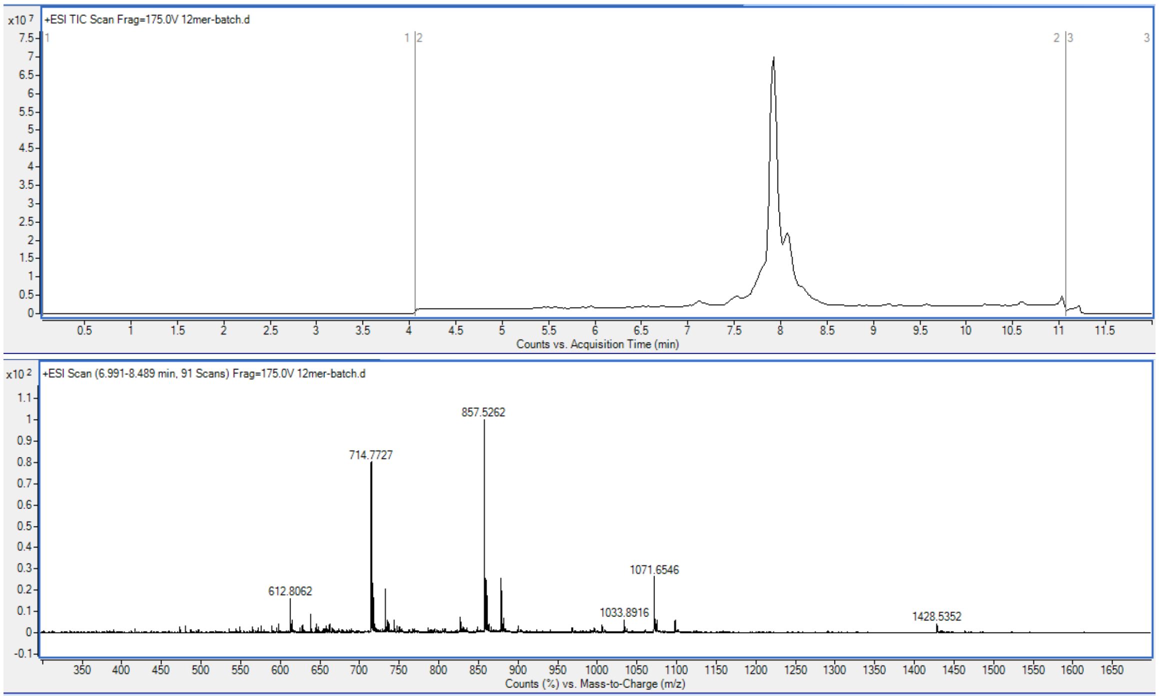

13.2.2 Tiny Tides synthesis of 12-mer PMO at 70˚C

Sequence (5’ to 3’): Tail-ACGTACGTACGT (12-mer)
Condition: Tiny Tides synthesis, 70 °C, 4-cypy·TFA, NEM neutr, DIEA base, LiBr 0.2 M, 18 eq monomer, 2.3-hour synthesis.
LCMS Method: **Condition 1**
Observed: 4280.64 Da Calculated: 4280.52 Da

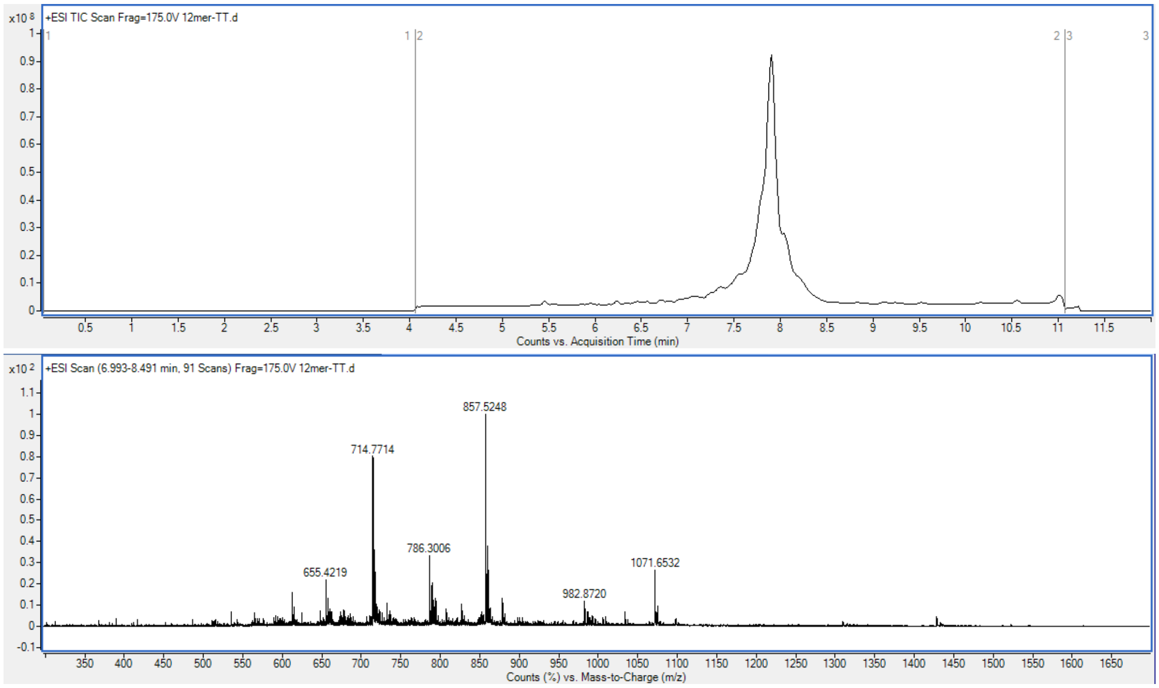

13.3 LCMS data of 18-mer (IVS2-654) 90˚C synthesis

The same 18-mer PMO was synthesized using both a batch protocol and rapid flow synthesis. Cleavage was performed using **Supplementary Section 4 method 2** for both samples. Purification of both samples was performed using **Supplementary Section 5.1 Method 2.**

13.3.1 Batch synthesis of 18-mer PMO (IVS2-654) (crude)

Sequence (5’ to 3’): Tail-GCTATTACCTTAACCCAG (18-mer)
Condition: Batch synthesis (**Supplementary Section 3**), 1-week synthesis.
LCMS Method: **Condition 3**
Observed: 6209.23 Da Calculated: 6209.19 Da

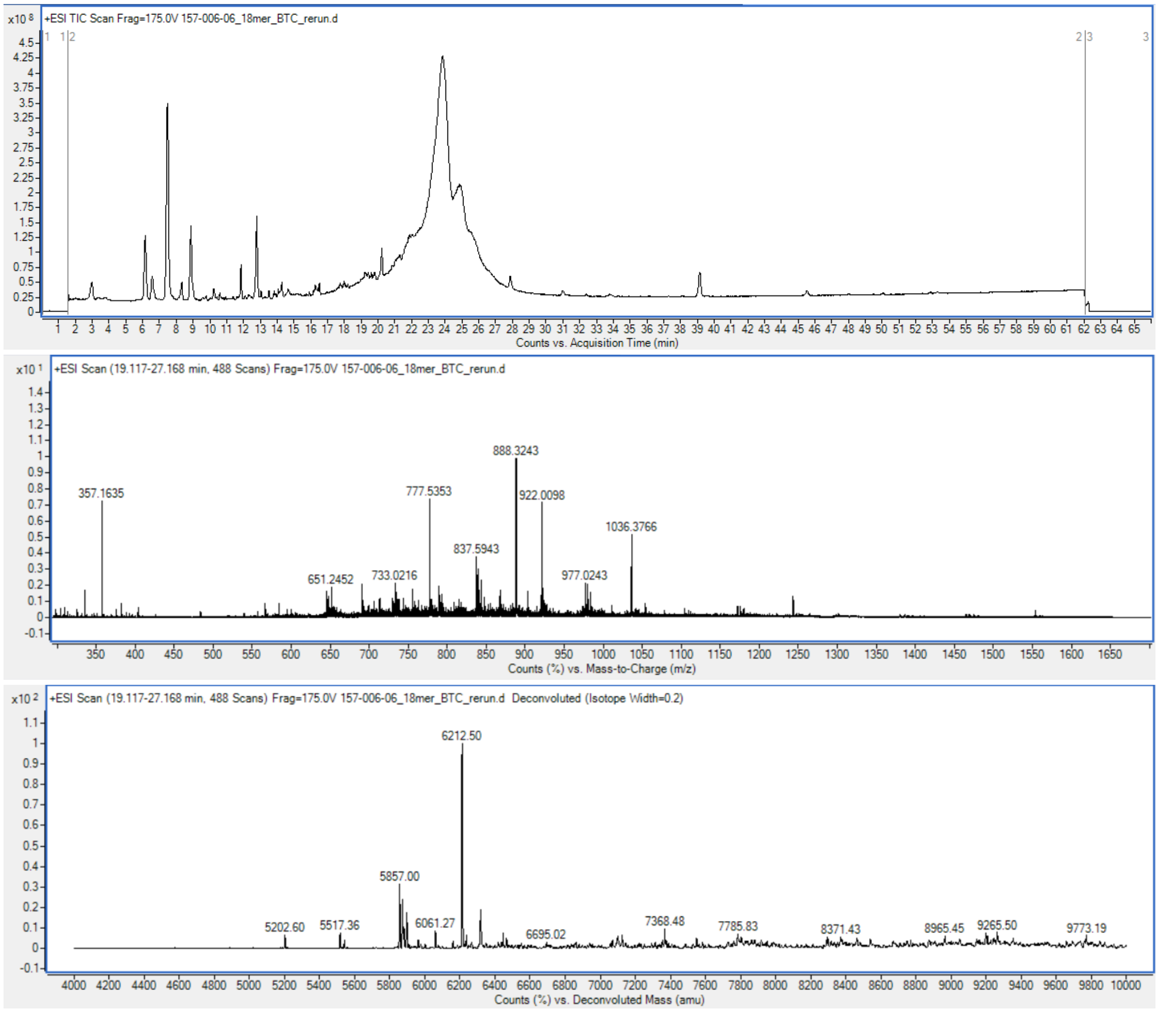

13.3.2 Batch synthesis of 18mer PMO (IVS2-654) (post purification)

Sequence (5' to 3'): Tail-GCTATTACCTTAACCCAG (18-mer)
Condition: Batch synthesis (**Supplementary Section 3**), 1-week synthesis.
LCMS Method: **Condition 3**
Observed: 6209.22 Da Calculated: 6209.19 Da

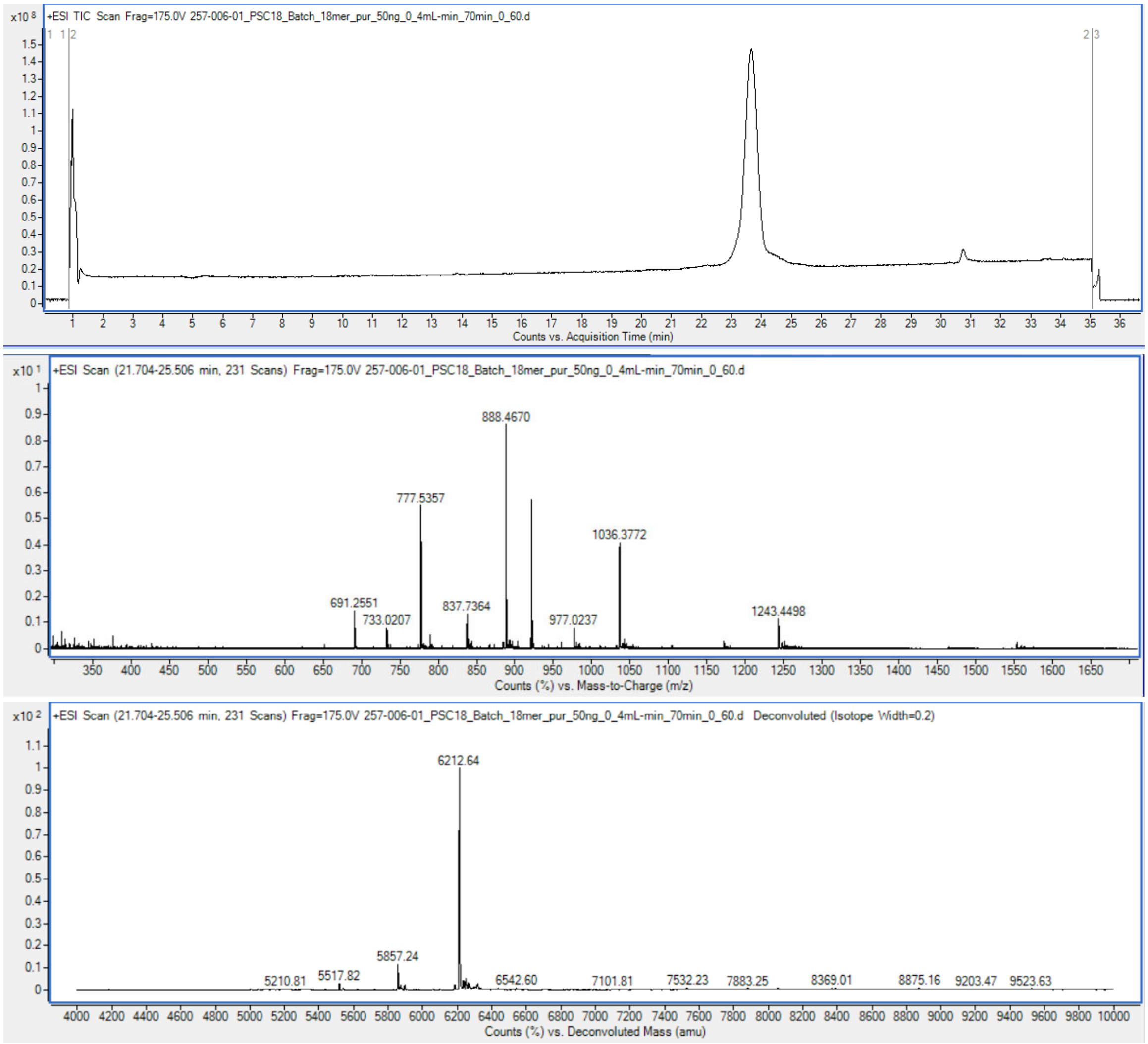

13.3.3 Tiny Tides synthesis of 18-mer PMO (IVS2-654) at 90˚C

Sequence (5' to 3'): Tail-GCTATTACCTTAACCCAG (18-mer)
Condition: Tiny Tides synthesis, 90 °C, 3,5-lut·TFA, NEM neutr, DIEA base, LiBr 0.2 M, 18 eq monomer, 3.5 hours.
LCMS Method: **Condition 3**
Observed: 6209.23 Da Calculated: 6209.19 Da

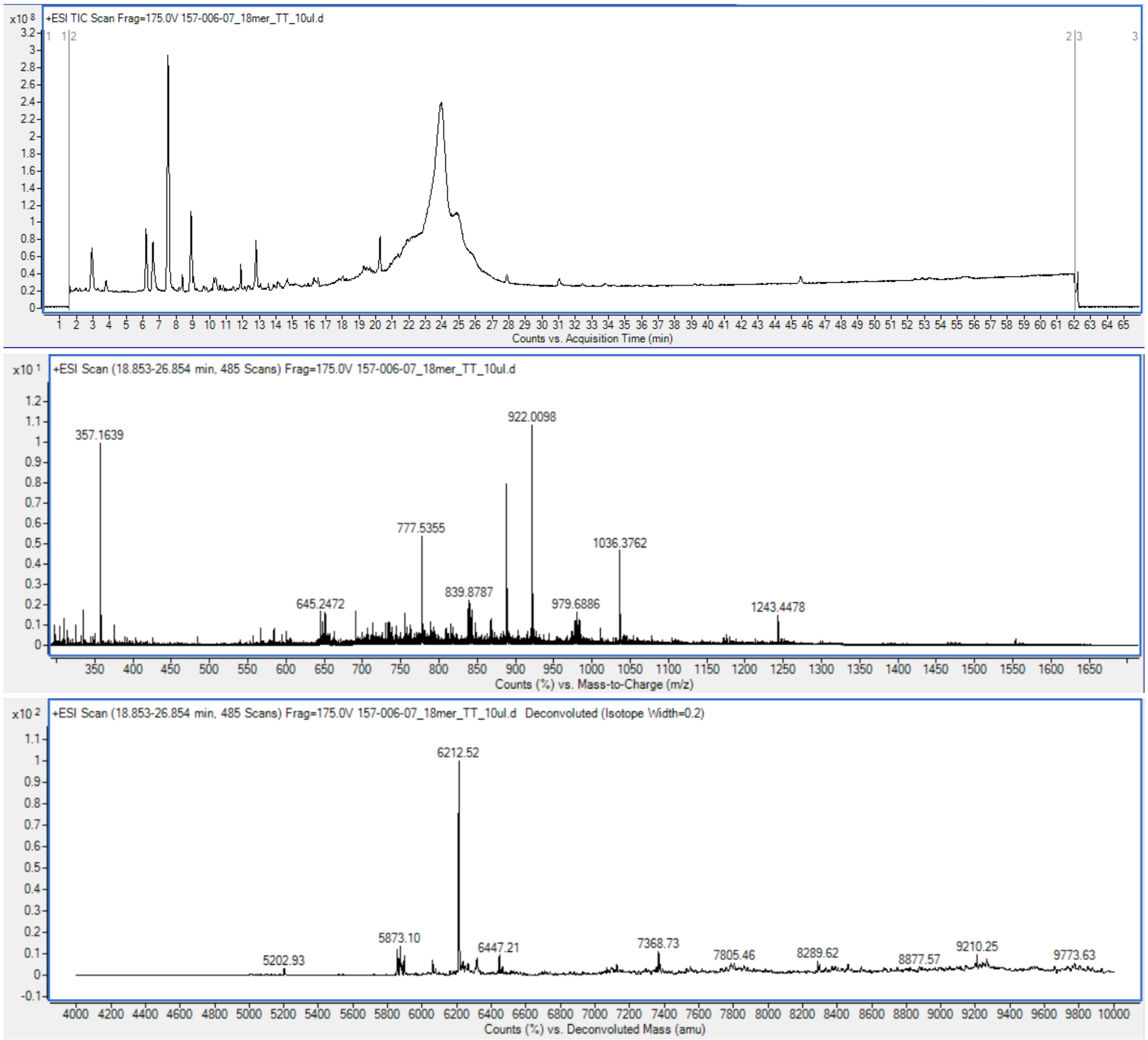

13.3.4 Tiny Tides synthesis of 18-mer PMO (IVS2-654) at 90˚C (post purification)

Sequence (5' to 3'): Tail-GCTATTACCTTAACCCAG (18-mer)
Condition: Tiny Tides synthesis, 90 °C, 3,5-lut·TFA, NEM neutr, DIEA base, LiBr 0.2 M, 18 eq monomer, 3.5 hours.
LCMS Method: **Condition 2**
Observed: 6209.20 Da Calculated: 6209.19 Da

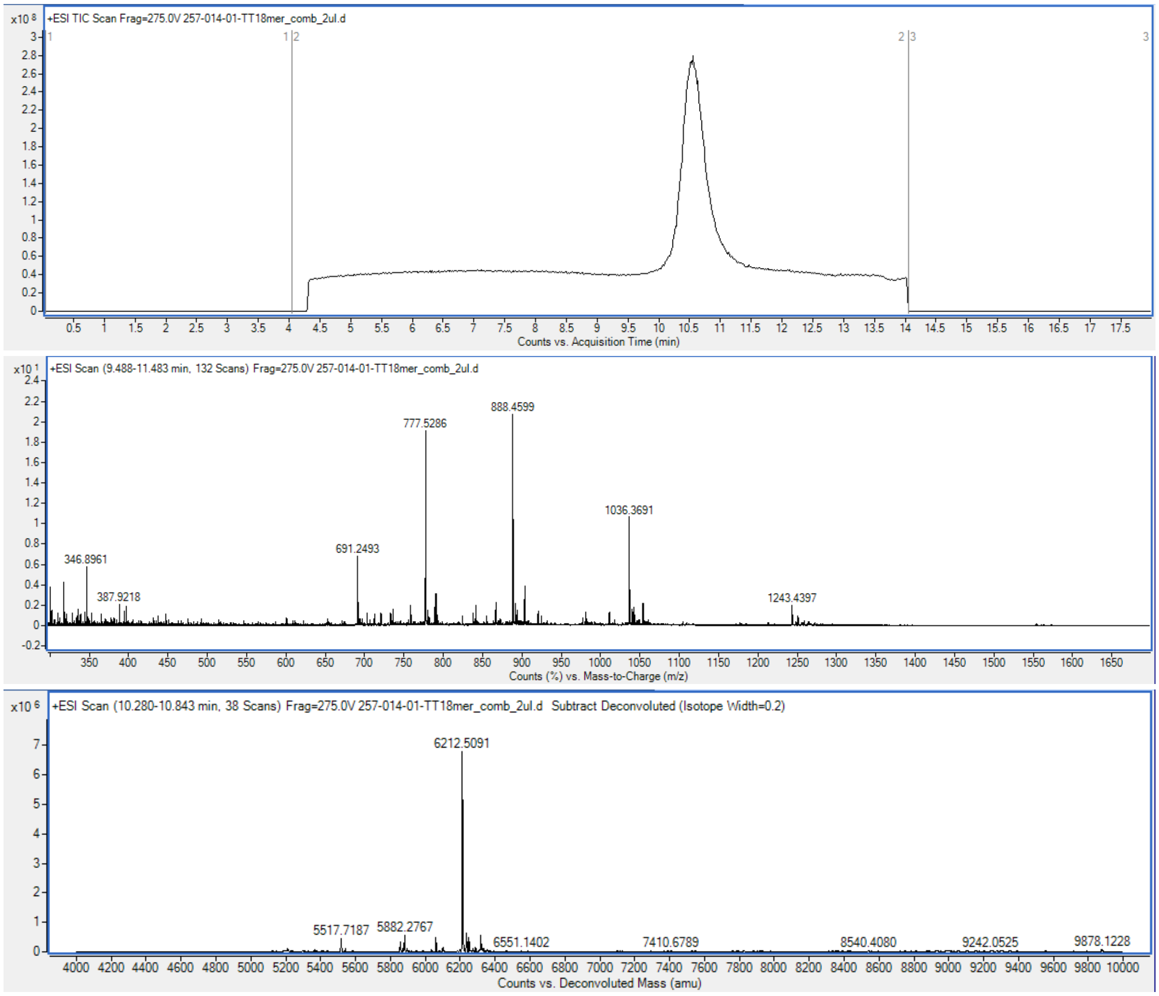

13.4 LCMS data of exon 46 targeted 20-mer sequences

Three PMO sequences designed to skip exon 46 were synthesized using Tiny Tides. Starting from 10 mg of resin, after synthesis and purification, 1 mg of purified 20-mer sample was obtained for each sequence. Purification was performed in two stages using methods **Supplementary Section 5.1 Method 1** and **Supplementary Section 5.2**. Cleavage was performed using **Supplementary Section 4 Method 2*.***

13.4.1 Tiny Tides synthesis of 20-mer PMO (seq. 1) at 90˚C

Sequence (5’ to 3’): Tail-CTTTTCTTTTAGTTGCTGCT (20-mer, seq. 1)
Condition: Tiny Tides synthesis, 90 °C, 3,5-lut·TFA, NEM neutr, DIEA base, LiBr 0.2 M, 18 eq monomer, 4.2 hours.
LCMS Method: **Condition 2**
Observed: 6887.37 Da Calculated: 6887.38 Da

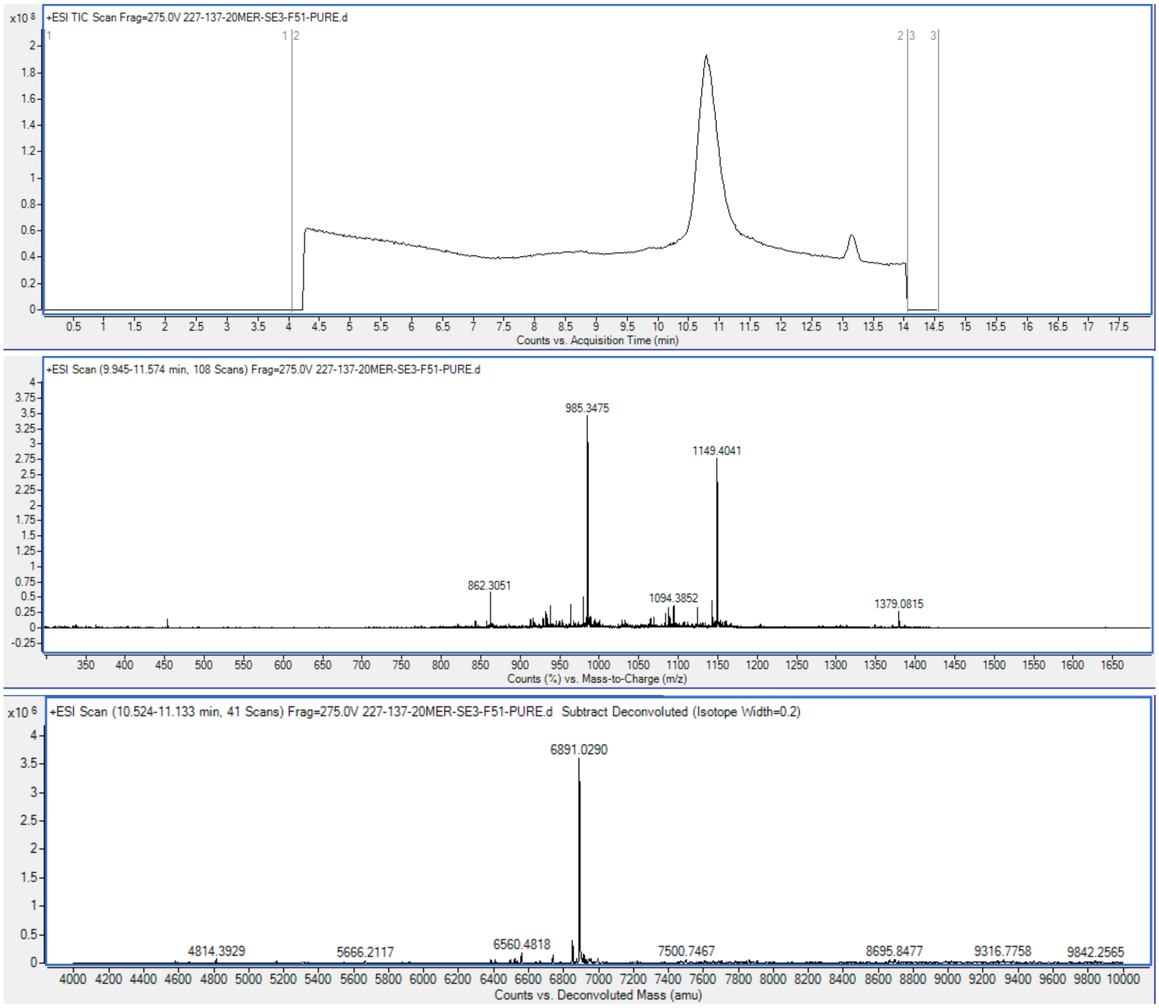

13.4.2 Tiny Tides synthesis of 20-mer PMO (seq. 2) at 90˚C

Sequence (5’ to 3’): Tail-CTGCTCTTTTCCAGGTTCAA (20-mer, seq. 2)
Condition: Tiny Tides synthesis, 90 °C, 3,5-lut·TFA, NEM neutr, DIEA base, LiBr 0.2 M, 18 eq monomer, 4.2 hours. Note that peaks at 9 and 13 mins are included in the background.
LCMS Method: **Condition 2**
Observed: 6876.45 Da Calculated: 6876.40 Da

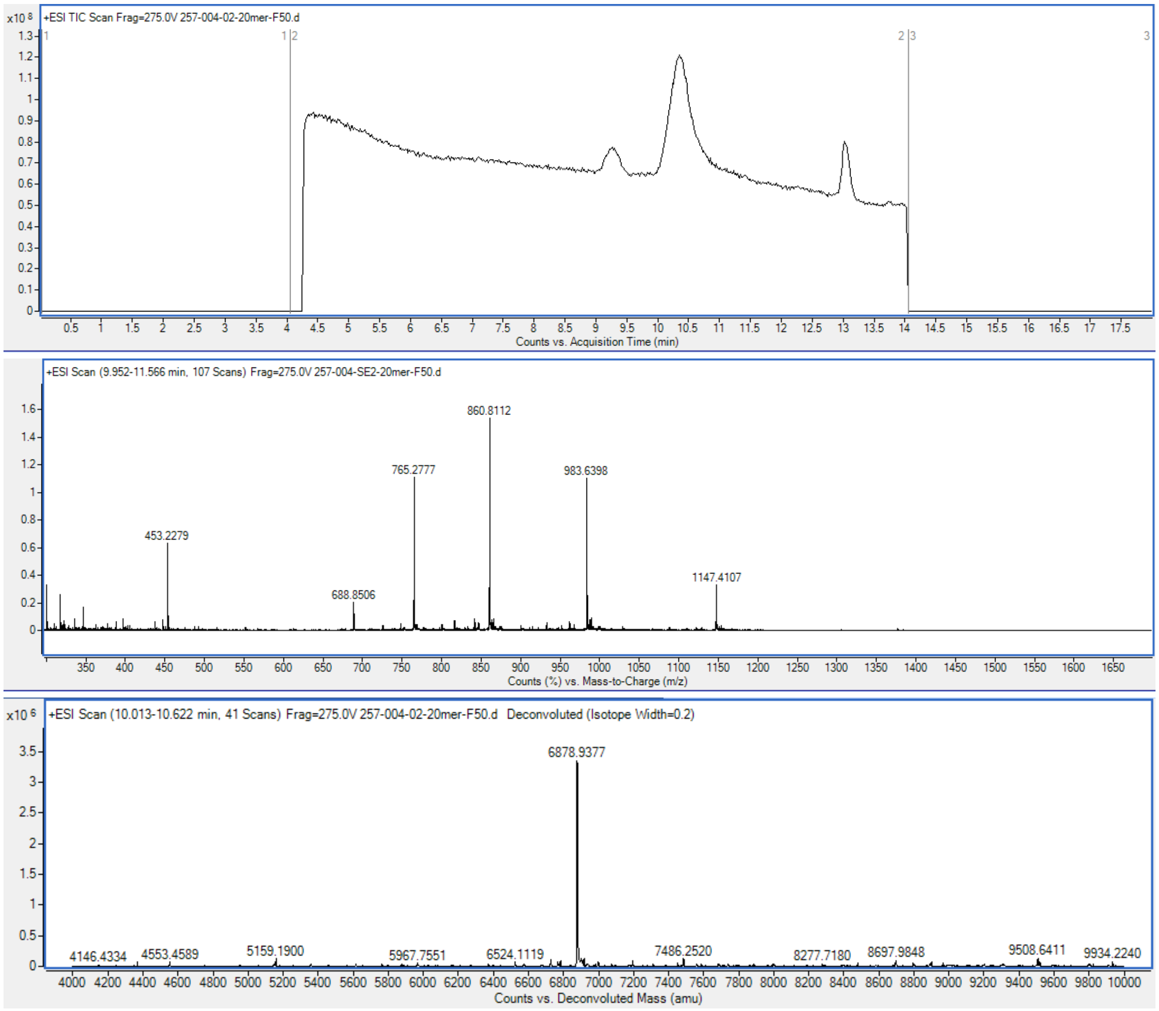
+ESI TIC Scan Frag=275.0V 257-004-02-20mer-F50.d
Counts vs. Acquisition Time (min)
+ESI Scan (9.952-11.566 min, 107 Scans) Frag=275.0V 257-004-SE2-20mer-F50.d
453.2279
688.8506
765.2777
860.8112
983.6398
1147.4107
Counts (%) vs. Mass-to-Charge (m/z)
+ESI Scan (10.013-10.622 min, 41 Scans) Frag=275.0V 257-004-02-20mer-F50.d Deconvoluted (Isotope Width=0.2)
6878.9377
4146.4334
4553.4589
5159.1900
5967.7551
6524.1119
7486.2520
8277.7180
8697.9848
9508.6411
9934.2240
Counts vs. Deconvoluted Mass (amu)

13.4.3 Tiny Tides synthesis of 20-mer PMO (seq. 3) at 90˚C

Sequence (5' to 3'): Tail- TTCTTTTGTTCTTCTAGCCT (20-mer, seq. 3)
Condition: Tiny Tides synthesis, 90 °C, 3,5-lut·TFA, NEM neutr, DIEA base, LiBr 0.2 M, 18 eq monomer, 4.2 hours.
LCMS Method: **Condition 2**
Observed: 6848.41 Da Calculated: 6848.37 Da

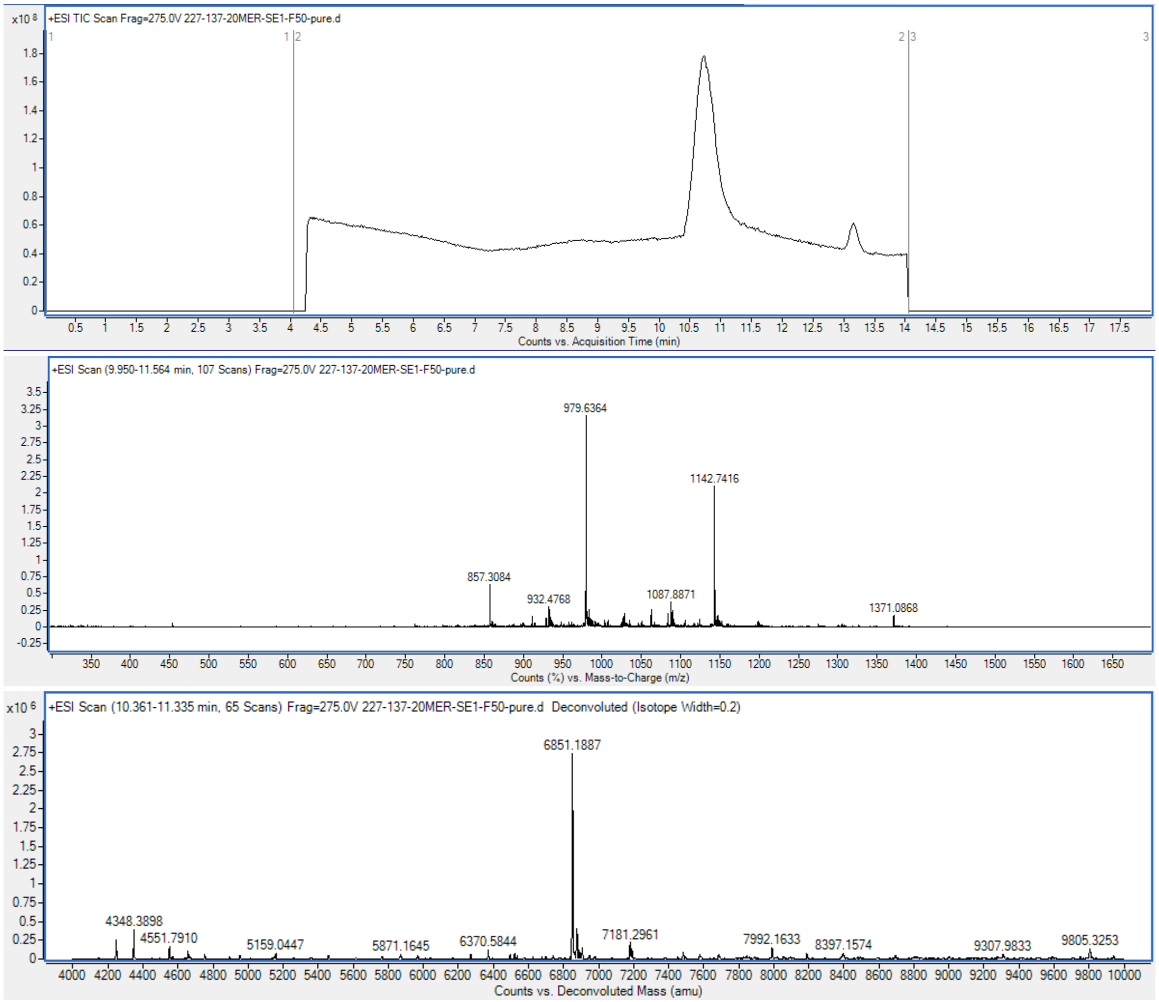

13.5 LCMS data of SARS-CoV-2 5'UTR TRS targeted sequence

A PMO sequence designed to bind the TRS in the 5'UTR of the SARS-CoV-2 genomic mRNA was synthesized using Tiny Tides. Purification was carried out with CEX then AEX in sequence using methods **Supplementary Section 5.1 Method 1 and Supplementary Section 5.3**.

Sequence (5' to 3'): Tail-TAAAGTTCGTTTAGAGAA (18-mer)
Condition: Tiny Tides synthesis, 90 °C, 3,5-lut·TFA, NEM neutr, DIEA base, LiBr 0.2 M, 20 eq monomer, 3.5 hours.
LCMS Method: **Condition 2**
Observed: 6352.22 Da Calculated: 6352.23 Da

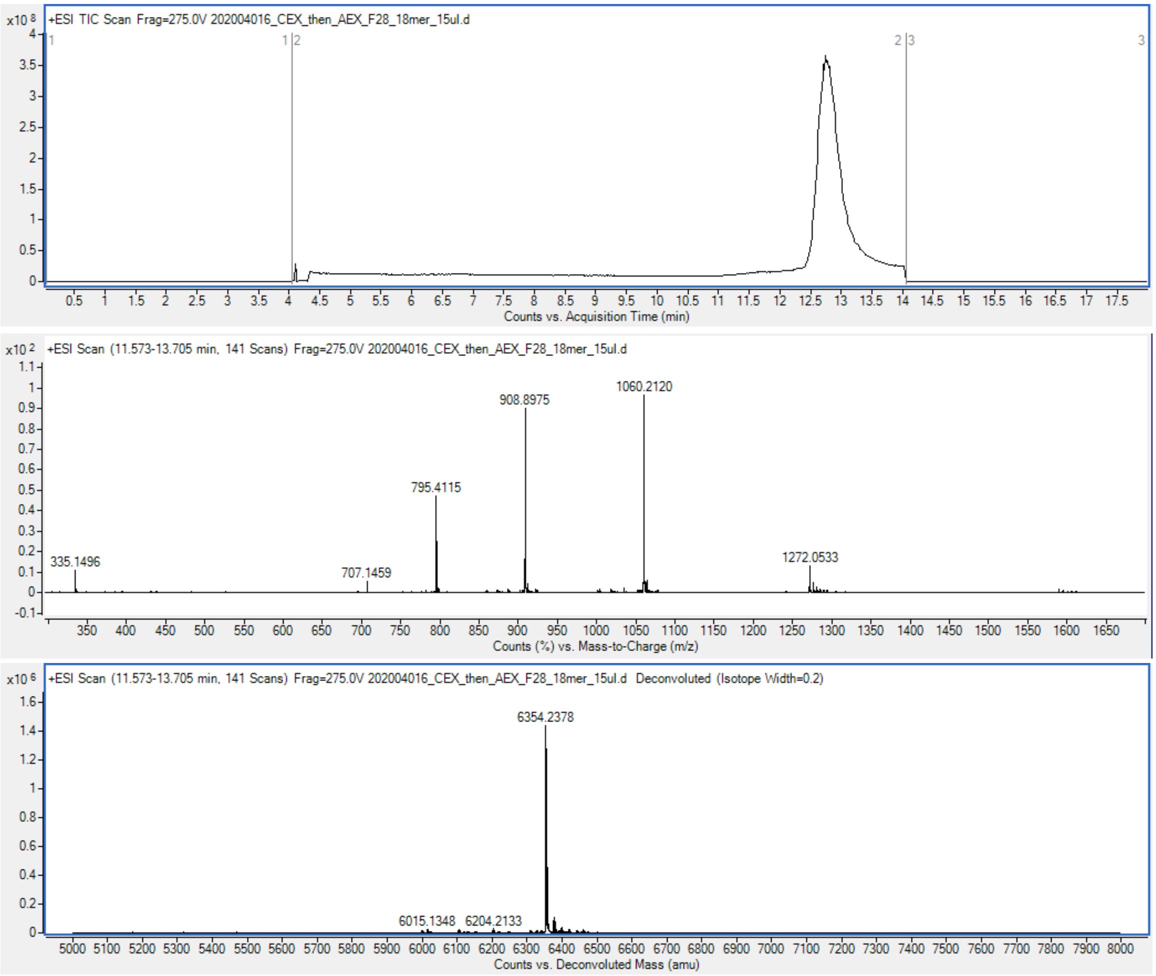

13.6 LCMS data of purified fusion inhibitor EK1

A previously reported SARS-CoV-2 fusion inhibitor, EK1, was used as a positive control for the virus infection assay. Peptide was synthesized on a automated peptide synthesizer and purified with Biotage.

EK1 amino acid sequence: SLDQINVTFLDLEYEMKKLEEAIKKLEESYIDLKEL
Synthesis condition: automated peptide synthesis, 90 °C, HATU (or PyAOP), DIEA base, 20% Piperidine, 28 eq monomer, 1.5 hours.
Purification condition: Biotage Sfär C18 D column (Duo 100 Å 30 µm, 12g), 5 to 60% acetonitrile over 12 column volume.
LCMS Method: Agilent Zorbax 300SB-C3 column, gradient: 1 to 91% acetonitrile over 15 mins.
Observed: 4328.17 Da Calculated: 4328.26 Da

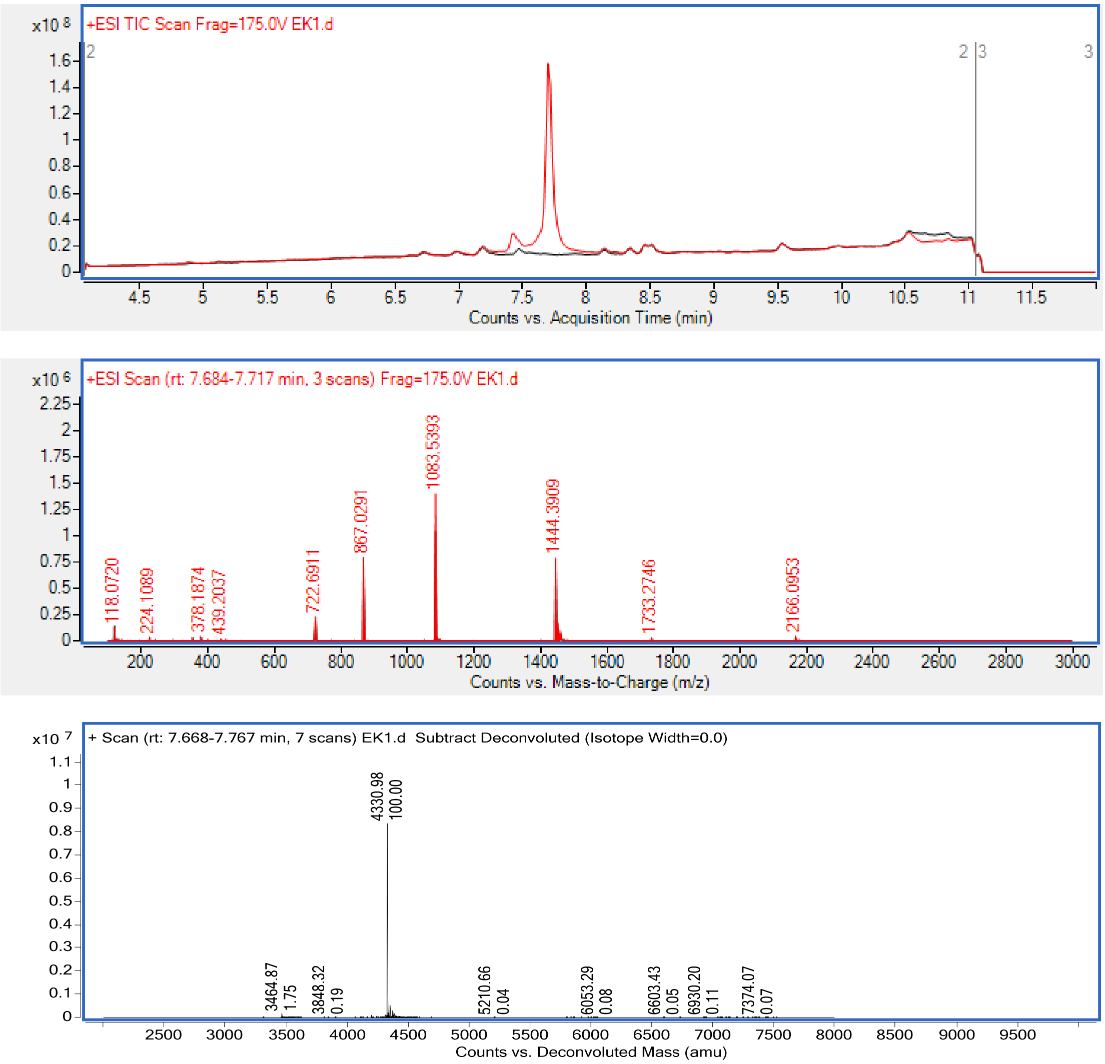

14. References

1. Fox, C. M. J., and Weller, D. D. (2008). Method of synthesis of morpholino oligomers. *US Pat.* US8299206.
2. Summerton, J., and Weller, D. (1997). Morpholino antisense oligomers: design, preparation, and properties. Antisense Nucleic Acid Drug Dev. *7*, 187-195.
3. Summerton, J. E., and Weller, D. D. (1991). Uncharged morpolino-based polymers having phosphorous containing chiral intersubunit linkages. US patent US5185444.
4. Harakawa, T., Tsunoda, H., Ohkubo, A., Seio, K., and Sekine, M. (2012). Development of an efficient method for phosphorodiamidate bond formation by using inorganic salts. Bioorg. Med. Chem. Lett. *22*, 1445-1447.
5. Bhadra, J., Pattanayak, S., and Sinha, S. (2015). Synthesis of morpholino monomers, chlorophosphoramidate monomers, and solid-phase synthesis of short morpholino oligomers. Curr. Protoc. Nucleic Acid Chem. *62*, 4.65.1-4.65.26.
6. Fox, C. M. J., and Weller, D. D. (2008). Method of synthesis of morpholino oligomers. US patent 8299206 B2.
7. Porter, R. S., and Johnson, J. F. (1970) Analytical calorimetry: proceedings of the symposium on analytical calorimetry at the meeting of the american chemical society (Springer).
8. Grandberg, I. I., Faizova, G. K., and Kost, A. N. (1967). Comparative basicities of substituted pyridines and electronegativity series for substituents in the pyridine series. Chem. Heterocycl. Compd. *2*, 421-425.
9. Clarke, K. & Rothwell, K. (1960) A kinetic study of the effect of substituents on the rate of formation of alkylpyridinium halides in nitromethane solution. J. Chem. Soc. *0* 1885-1895.
10. Benjamin, Lee, amijalis, littleblackfish, & NLPohl. (2020, April 28). a-callahan/MechWolf_Pull 0.1.1 (Version 0.1.1). Zenodo. http://doi.org/10.5281/zenodo.3774509.
11. Fogler, H. S. (2016) Elements of chemical reaction engineering, fifth edition (Prentice Hall).
12. Levenspiel, O. (1998) Chemical reaction engineering, third edition (Wiley).
13. Simon, M. D., Heider, P. L., Adamo, A., Vinogradov, A. A., Mong, S. K., Li, X., Berger, T., Policarpo, R. L., Zhang, C., Zou, Y., et al. (2014). Rapid flow-based peptide synthesis. ChemBioChem *15*, 713-720.
14. Nagy, K. D., Shen, B., Jamison, T. F., and Jensen, K. F. (2012). Mixing and dispersion in small-scale flow systems. Org. Pro. Res. & Devel. *16*, 976-981.
