# Supplementary material for: Fully automated fast-flow synthesis of antisense phosphorodiamidate morpholino oligomers
Source: Nat Commun. 2021 Jul 20;12:4396. doi: 10.1038/s41467-021-24598-4 (PMC8292409; doi:10.1038/s41467-021-24598-4)
Supplement: Supplementary file 3 — Reporting Summary [file 41467_2021_24598_MOESM3_ESM.pdf]

nature research

Corresponding author(s): Bradley L Pentelute

Last updated by author(s): May 18, 2021

Reporting Summary

Nature Research wishes to improve the reproducibility of the work that we publish. This form provides structure for consistency and transparency in reporting. For further information on Nature Research policies, see our Editorial Policies and the Editorial Policy Checklist.

Statistics

For all statistical analyses, confirm that the following items are present in the figure legend, table legend, main text, or Methods section.

n/a	Confirmed	
☐	☒	The exact sample size (*n*) for each experimental group/condition, given as a discrete number and unit of measurement
☐	☒	A statement on whether measurements were taken from distinct samples or whether the same sample was measured repeatedly
☒	☐	The statistical test(s) used AND whether they are one- or two-sided<br>*Only common tests should be described solely by name; describe more complex techniques in the Methods section.*
☒	☐	A description of all covariates tested
☒	☐	A description of any assumptions or corrections, such as tests of normality and adjustment for multiple comparisons
☒	☐	A full description of the statistical parameters including central tendency (e.g. means) or other basic estimates (e.g. regression coefficient) AND variation (e.g. standard deviation) or associated estimates of uncertainty (e.g. confidence intervals)
☒	☐	For null hypothesis testing, the test statistic (e.g. *F*, *t*, *r*) with confidence intervals, effect sizes, degrees of freedom and *P* value noted<br>*Give P values as exact values whenever suitable.*
☒	☐	For Bayesian analysis, information on the choice of priors and Markov chain Monte Carlo settings
☒	☐	For hierarchical and complex designs, identification of the appropriate level for tests and full reporting of outcomes
☒	☐	Estimates of effect sizes (e.g. Cohen's *d*, Pearson's *r*), indicating how they were calculated

Our web collection on statistics for biologists contains articles on many of the points above.

Software and code

Policy information about availability of computer code

Data collection	Agilent MassHunter (Version B.06.01), Bruker FlexControl (Version 3.4, Build 135), MechWolf (http://doi.org/10.5281/zenodo.3774509), Python (Version 2.7.17)
Data analysis	Agilent MassHunter (Version B.06.00, Service Pack 1), Bruker FlexAnalysis (Version 3.4, Build 76)

For manuscripts utilizing custom algorithms or software that are central to the research but not yet described in published literature, software must be made available to editors and reviewers. We strongly encourage code deposition in a community repository (e.g. GitHub). See the Nature Research guidelines for submitting code & software for further information.

Data

Policy information about availability of data

All manuscripts must include a data availability statement. This statement should provide the following information, where applicable:

- Accession codes, unique identifiers, or web links for publicly available datasets
- A list of figures that have associated raw data
- A description of any restrictions on data availability

All the data generated during this study are included in the published article (and in the Supplementary Information). Further details are available from the corresponding authors upon request.

Field-specific reporting

Please select the one below that is the best fit for your research. If you are not sure, read the appropriate sections before making your selection.

- [x] Life sciences
- [ ] Behavioural & social sciences
- [ ] Ecological, evolutionary & environmental sciences

For a reference copy of the document with all sections, see nature.com/documents/nr-reporting-summary-flat.pdf

Life sciences study design

All studies must disclose on these points even when the disclosure is negative.

Sample size	Sample size for synthetic experiments was not predetermined. The sample size for automated synthesis consisted of a total of 8 PMO sequences of increasing length from 4 to 20 residues. For stability experiments, we performed 9 studies, corresponding to either 3 or 4 different conditions for the PMO sequences selected for the measurements, leading to a total sample size of 31 experiments.<br>For the SARS-CoV-2 cell assays, we performed 2 biological replicates with 2 technical repeats each. Together, all the samples fit on the same plate for a given biological replicate so that each sample is subjected to the same PCR reagents and conditions. Additional sample sizes with the dilution conditions would push some off onto another plate to run at another time rather than with the bulk of the samples. This could create errors from plate to plate as well as pipetting errors. If the cycle thresholds (CTs) were outside of 10% for comparable wells, additional qPCRs (up to 4) would be run. We also used duplicates since our quantification cycle (Cq) was less than 0.5 and our system flagged no errors.
Data exclusions	All data are included for each experiment described. No data were excluded from the analyses. Additionally, for the SARS-CoV-2 assay, all measured CTs were within 10% of comparable wells, and accordingly all data were included in the analysis.
Replication	All PMO syntheses were carried out with technical replicates n >= 2 for each sequence sample. Stability data did not include technical replicates. PMO stability results were reconfirmed via the successful syntheses of long PMO sequences.<br><br>For the SARS-CoV-2 assay, all attempts in the second replicate were successful using the 10% CT deviance threshold. We were also using a 10% cutoff between each repeat to verify the data was clustering together between the biological and technical replicates. Additionally, our Cq was less than 0.5, and our system flagged no errors. Had we observed these effects, we would have run additional replicates to verify the respective data point.
Randomization	Randomization is not relevant in this study, because there are no random variables associated with synthetic purity that could be controlled by using such a study design.
Blinding	For the SARS-CoV-2 live inhibition assays, the researcher performing the experiments was not provided with the identity of the compounds tested and hence was blinded to the treatment groups. The returned RNA samples were coded by an assigned identifier by the blinded researcher and run by qRT-PCR. Only after the CTs were known were the sample identifiers unmasked and put into relevant sample columns based on sample identity (i.e., EK1 at 0.1 uM). No sample CT value was suppressed before or after they were given their sample IDs through unmasking and analyzed by Prism software for a meaningful difference from the positives.

Reporting for specific materials, systems and methods

We require information from authors about some types of materials, experimental systems and methods used in many studies. Here, indicate whether each material, system or method listed is relevant to your study. If you are not sure if a list item applies to your research, read the appropriate section before selecting a response.

Materials & experimental systems

n/a	Involved in the study	
x		Antibodies
	x	Eukaryotic cell lines
x		Palaeontology and archaeology
x		Animals and other organisms
x		Human research participants
x		Clinical data
x		Dual use research of concern

Methods

n/a	Involved in the study	
x		ChIP-seq
x		Flow cytometry
x		MRI-based neuroimaging

Eukaryotic cell lines

Policy information about cell lines

Cell line source(s)	Vero E6 cells: American Tissue Culture Collection
Authentication	Cells were not authenticated
Mycoplasma contamination	Cells were screened for mycoplasma using InVivogen's mycoplasma detection system, and tested negative.
Commonly misidentified lines (See ICLAC register)	No commonly misidentified cell lines were used in the study.
